# Molecular modeling simulation studies reveal new potential inhibitors against HPV E6 protein

**DOI:** 10.1371/journal.pone.0213028

**Published:** 2019-03-15

**Authors:** Joel Ricci-López, Abraham Vidal-Limon, Matías Zunñiga, Verónica A. Jimènez, Joel B. Alderete, Carlos A. Brizuela, Sergio Aguila

**Affiliations:** 1 Centro de Nanociencias y Nanotecnología, Universidad Nacional Autonoma de Mèxico, Ensenada, Baja California, México; 2 Departamento de Ciencias Químicas, Facultad de Ciencias Exactas, Universidad Andres Bello, Sede Concepción, Chile; 3 Departamento de Química Orgánica, Facultad de Ciencias Químicas, Universidad de Concepción, Concepción, Chile; 4 Instituto de Química de Recursos Naturales, Universidad de Talca, Talca, Chile; 5 Computer Science Department, CICESE Research Center, Ensenada, Mèxico; Universita degli Studi di Torino, ITALY

## Abstract

High-risk strains of human papillomavirus (HPV) have been identified as the etiologic agent of some anogenital tract, head, and neck cancers. Although prophylactic HPV vaccines have been approved; it is still necessary a drug-based treatment against the infection and its oncogenic effects. The E6 oncoprotein is one of the most studied therapeutic targets of HPV, it has been identified as a key factor in cell immortalization and tumor progression in HPV-positive cells. E6 can promote the degradation of p53, a tumor suppressor protein, through the interaction with the cellular ubiquitin ligase E6AP. Therefore, preventing the formation of the E6-E6AP complex is one of the main strategies to inhibit the viability and proliferation of infected cells. Herein, we propose an *in silico* pipeline to identify small-molecule inhibitors of the E6-E6AP interaction. Virtual screening was carried out by predicting the ADME properties of the molecules and performing ensemble-based docking simulations to E6 protein followed by binding free energy estimation through MM/PB(GB)SA methods. Finally, the top-three compounds were selected, and their stability in the E6 docked complex and their effect in the inhibition of the E6-E6AP interaction was corroborated by molecular dynamics simulation. Therefore, this pipeline and the identified molecules represent a new starting point in the development of anti-HPV drugs.

## Introduction

Human papillomavirus (HPV) infection is one of the most common sexually transmitted diseases. Due to their oncogenic effect, some of the HPV strains have been identified as high-risk (HR) types, being the leading cause of cervical cancer and the etiologic agent of some anogenital tract and head and neck cancers [1]. Epidemiologically, HPV-16 is the most prevalent type in cervical cancer, accounting for approximately 55% of all cases [2]. Nowadays prophylactic vaccines, *Cervarix* [3] and *Gardasil* [4], have been approved and effectively applied for the prevention of HPV infection. However, for people already infected, current therapies consist of the use of chemotherapeutic agents or the application of surgical and ablative techniques to eliminate developed tumors [5]. These treatments are invasive, non-specific, and tend to be expensive, difficulting their availability to millions of patients, particularly in developing countries. Hence, one of the main alternatives to treat HPV-related diseases is the development of accessible drug-based therapies directed against the virus.

The E6 and E7 proteins, encoded by HPVs, take control of the cell cycle regulatory functions and promote the proliferation of infected keratinocytes. Nevertheless, in HR HPVs types the continuous expression of both proteins leads to genomic instability, which plays a critical role in the cellular transformation and tumorigenesis [6]. E7 mediates the degradation of Retinoblastoma (pRb) family members promoting the S-phase progression. As a result, HPV genome replication is promoted, and a collateral cellular DNA damage and chromosomal abnormalities can be produced [7]. Under normal situations, cells with genomic instability are targeted by p53 for cell cycle arrest or apoptosis. However, E6 protein ensures cell immortalization by forming a complex with the cellular E3 ligase E6-associated protein (E6AP) and targeting p53 for degradation via the ubiquitin-proteasome pathway [6, 8].

HPV-16 E6 is a small protein of 158 residues featuring two Zn^2+^ binding domains joined by a helix linker of 36 amino acids [8]. Despite its size, E6 can bind to multiple cellular proteins through a PDZ-domain-binding motif or by an inter-domain groove that acts as a *LxxLL* binding pocket [8]. In the case of E6-E6AP interaction, E6 pocket recognizes the *LxxLL* helical motif of the HECT domain of E6AP, which in turn recruits p53 to establish the p53 degradation complex [9]. Since HPV-induced tumors contain high levels of nonmutated p53 [10], the disruption E6-E6AP interaction is a promising therapeutic strategy that focuses on the reactivation of p53 protein functions to ultimately induce cellular apoptosis of HPV-transformed cells. Besides, the E6 pocket poses a particular structure that cellular *LxxLL*-binding proteins do not have [11]. This structural difference can be exploited to improve binding selectivity against a viral protein with regards to cellular components. Thereby, E6 pocket protein is one of the major targets for drug development against HPV infection and its oncogenic effects.

Several studies have tested the inhibition of the E6-E6AP interaction through different molecules, including alpha helical peptides [12, 13], intrabodies [14], and small molecules [15–17]. Nevertheless, most molecules have shown moderate activity or low bioavailability. Therefore, the identification of pharmacologically active compounds to treat HPV is still necessary. However, drug development is a highly demanding process of economic and time resources, lasting longer than a decade and with an average cost of $2.8 billion per drug approved [18]. Computer-aided drug discovery (CADD) is an attractive complement to drug development, particularly in the early stages. CADD attempts to improve the efficacy of the hit discovery process through *in silico* methodologies by testing and screening a large number of compounds to identify a small group of candidates with desirable pharmacological properties. This approach made the drug discovery process more goal-oriented, saving resources in terms of time and money [19].

In the present study, we aim to identify new candidate compounds with favorable pharmacokinetics properties and capable of inhibiting the E6-E6AP interaction. To achieve this goal, an *in silico* analysis was performed through a pipeline comprised of absorption, distribution, metabolism, and excretion (ADME) properties prediction, Structural-based Virtual Screening (SBVS), and Molecular Dynamics (MD) simulations. Homology modeling and MD were used to obtain a set of HPV-16 E6 protein structures.

Then, Ensemble-based docking simulations were employed to evaluate a library of molecules structurally related to compounds that have shown anti-HPV activity. After rescoring with MM/GBSA and MM/PBSA methods, three candidate compounds were evaluated by MD in interactions with E6 protein. Two of these identified compounds appear to be promising candidates for future *in vitro* evaluations. Moreover, the data provided by the SBVS results, along with E6 protein dynamics evaluation, afford valuable information for continuing the development and optimization of new drugs against HR HPV infection and HPV-related cancer.

## Materials and methods

### Ligand selection and ADME filtering

Twenty-six small molecules were identified from the literature as active compounds against HPV-positive cells by *in vitro* assays, and/or against E6 protein by *in silico* approaches. These molecules, defined as “reference compounds”, were used as queries in the Zinc15 [20] database in order to obtain a chemical library with molecules structurally related, but without previously evaluated anti-HPV activity. Tanimoto and Dice similarity indices (index ≥ 0.6), along with the substructure search, were used to search for these compounds.

Subsequently, the compound library was retrieved in SMILES format and was processed with the LigPrep module of the Schr¨odinger suite [21] and with the OPLS3 force field [22] to generate 3D models with ionization states at pH 7.0 ± 0.2. The ADME properties were predicted using the QikProp module of the Schrödinger suite [23]. The ADME-compliance score drug-likeness parameter (*#stars*) was used to screen the compound library along with the next four descriptors: Lipinski’s rule of five [24], Jorgensen’s rule of three [25], human oral absorption and predicted skin permeability [26]. Only one violation per descriptor was permiHed.

### Protein preparation

Homology modeling was performed to get the full-length structure of the HPV-16 E6 protein. For this purpose, the HPV-16 E6 sequence query was obtained from UniProt (ID: P03126) [27], and the template was retrieved from the crystal structure of E6/E6AP/p53 complex (PDB code 4XR8, resolution 2.25 Å); a 4C/4S mutant and nonfull-sequence structure of E6 [9]. The homology model building was carried out using Modeller v9.15 [28], and the model with the lowest value of DOPE assessment score [29] was selected for further analysis. The protonation states of ionizable residues of the E6 model were assigned using PROPKA [30]. The ff14SB force field [31] was used to describe the parameters of the protein along with the Zinc Amber Force Field (ZAFF) [32] for the two (Cys)_4_Zn^2+^ fingers. Then, the system was neutralized with Cl^-^ counterions and solvated in a TIP3P water box extended 15 Å from any protein atom in all three dimensions.

### Molecular dynamics simulations

All-atom explicit-solvent molecular dynamics (MD) simulations were carried out on GPUs using the CUDA version of pmemd of Amber16 [33]. Three independent MD assays were performed both for the apo state of E6 (apo-E6 systems) and for the E6-*LxxLL* complex (E6-hx systems). The energy minimization of each system was accomplished by 5000 steps of steepest descent minimization followed by 5000 steps of conjugate gradient minimization. Next, a simulated annealing strategy was performed as follows: (i) During the annealing step, the system was gradually heated from 0 K to 300 K in 0.5 ns. (ii) The temperature was maintained at 300 K for 0.5 ns. (iii) The temperature was increased to 400 K in 0.5 ns. (iv) The temperature was maintained at 400 K for 0.5 ns. (v) The system was cooled to 310 K in 0.5 ns. (vi) The system was equilibrated at 310 K for 5.5 ns.

During the process described above, calculations were performed in an NVT ensemble with periodic boundary conditions, and with an integration time step of 1 fs. Covalent bonds to hydrogen atoms were constrained by the SHAKE algorithm [34]. Long-range electrostatic interactions were handled using the particle mesh Ewald summation method, with a cutoff distance radius of 10 Å. Root-mean-square deviation (RMSD) of C*α* atoms was evaluated through time to assess the system equilibration. Finally, 100 ns of MD production was carried out in an NPT ensemble at 310 K and 1 bar using the Langevin thermostat and the isotropic position scaling, respectively. The time step used was 2 fs, and coordinate trajectories were saved every 20 ps.

### Trajectory analysis

The trajectory analysis was carried out using VMD software [35] implementations and the Bio3D package [36] for R [37]. 5000 snapshots were extracted from the 100 ns of each MD production. RMSD and Root-mean-square fluctuation (RMSF) values were calculated considering the C*α* atoms of the protein. Both measurements were used to analyze the conformational evolution of the protein and to highlight its flexible regions, respectively. Additionally, the volume of the *LxxLL* binding pocket was measured employing the POVME 3.0 tool [38]. For apo-E6 systems, Principal Component Analysis (PCA) was performed over cartesian coordinates of C*α* atoms of all snapshots in the trajectories, and considering both the total sequence of E6 and only the residues corresponding to the pocket. Each trajectory was projected onto the first eigenvectors subspaces to reveal concerted atomic displacements, representing the “essential dynamics” of the protein [39].

To identify representative conformations of apo-E6, a clustering analysis was applied over each trajectory described by the first three principal components (PCs). Hierarchical clustering was carried out using the *hclust* function from R’s *stats* package [37]. The number of clusters in each trajectory was determined by visualizing the cluster dendrogram. Finally, the structure with the lowest RMSD value compared with the average structure of each cluster was selected as the representative conformation of that cluster.

### Structure-based virtual screening

Two stages of Structure-Based Virtual Screening (SBVS) were carried out to evaluate the set of molecules filtered according to their ADME profile. Hereinafter, the term “ligands” will be used to refer to these molecules. For the first SBVS stage, we conducted an ensemble docking strategy by docking the ligands into each of the apo-E6 conformations obtained by MD. For this purpose, we employed the Autodock Vina (Vina) software package [40].

The python scripts provided by AutoDockTools [41] were used to prepare the ligands and receptor pdbqt files, adding polar hydrogen atoms and Gasteiger charges. The docking grid size was 21x21x21 Å^3^, encompassing the entire *LxxLL* binding pocket. An exhaustiveness value of 64 was used, keeping the rest of the parameters with their default values. The best docking pose, as judged by the Vina score, was selected for each ligand in every E6 confirmation. Then, for each apo-E6 conformer, a set of ligands with the lowest free-binding energies was chosen. Finally, the ligands intersected in all sets were selected for further evaluation.

For the second SBVS stage, we used only one apo-E6 conformation, which corresponded to the most populated conformational cluster. The docking simulations were performed in Autodock 4.2 [41] (AD4), using a docking grid of 21x21x21 Å^3^, and employing the Lamarckian genetic algorithm, with 50 independent runs for each ligand.

Each population consisted of 150 individuals, with a mutation rate of 0.02, a maximum number of 2.7x10^4^ generations and a maximum number of 5.0x10^7^ energy evaluations.

### MM/PBSA and MM/GBSA methods

MM/PBSA and MM/GBSA methods were employed to estimate the binding free energies (Δ*H*_bind_) of each of the 100 top-ranked E6-ligand (E6-lig) complexes calculated by AD4, using Eq 1:

$$\Delta H_{bind}=\Delta H_{complex}\;-\;\Delta H_{receptor}\;-\;\Delta H_{ligand}$$ (1)

The corresponding free energy Δ*H* of each molecule was decomposed as follows (Eq 2):

$$\Delta H_{molecule}\;=\;\Delta EMM\;+\;\Delta H_{solv}\;-\;T\;\Delta S$$ (2)

$$\Delta H_{solv}\;=\;\Delta H_{polar}\;(PB\;or\;GB)\;+\;\Delta H_{non-polar}\;(SA)$$ (3)

where Δ*E*_*MM*_ is the change of molecular mechanical gas-phase energies, Δ*H*_*solv*_ is the polar and nonpolar solvation free energy, and *T* Δ*S* is the conformational entropy contribution to the free energy. For Δ*H*_solv_ (Eq 3), the polar contribution was computed by solving the generalized Born (GB) or Poisson-Boltzmann (PB) equation, and the nonpolar term was determined as a function of the solvent-accessible surface area. The entropic contribution was neglected due to its controversial benefit to Δ*H*_bind_ estimation, usually adding a significant random scattering to results [42]. Thus, it was assumed that *T* Δ*S* was similar across the evaluated E6-lig complexes [43]. The calculations of energy components in Eqs. 1–3 were carried out using the MMPBSA.py script [44] implemented in Amber16. For GB calculations the OBC model [45] was used (igb = 2) with a salt concentration of 0.15 M. The PB calculations were performed using an ionic strength of 0.15 M and the Amber default atom-type/charge-based radii. The rest of parameters were kept as default. The 100 top-ranked ligands, according to AD4 score, were selected for Δ*H*_bind_ calculation. This was accomplished by using the Antechamber suite to determine the atom types, partial charges and force field parameters of each ligand utilizing the GAFF force field [46]. Then, each E6-lig docked conformation obtained by AD4 was subjected to energy minimization in explicit solvent followed by rescoring using MM/PB(GB)SA. Finally, using a Pareto-based ranking scheme [47], we selected the optimal ligands concerning to their Δ*H*_bind_ values obtained by MM/PBSA and MM/GBSA. That is, out of all solutions, we selected the set of non-dominated ones, i.e., solutions that are not improved in both Δ*H*_bind_ terms by any other solution.

### MD analysis of the best candidate compounds

E6-lig complexes corresponding to non-dominant solutions were evaluated through MD, using the same protocol described for the apo-E6 protein. Additionally, the compound luteolin was selected to work as a positive control to contrast the results of the three candidate compounds. Thus, the E6-luteolin complex was also evaluated. As a first step, each E6-lig complex was subjected to 50 ns of MD. For the second step, the *LxxLL* helical motif of E6AP was docked to each E6-lig complex. Specifically, the helix (hx) was placed 12.3 Å from the center of mass of E6 protein in a similar position as that of the 4XR8 model, but avoiding overlap with the atoms of the ligand docked to E6. Then, 50 ns of MD were carried out for these [E6-lig]-hx complexes. Additionally, for positive control, an [E6]-hx system was simulated having the helix in the same position that the [E6-lig]-hx complexes, but with no ligand between E6 and the helical motif.

Finally, in both E6-lig and [E6-lig]-hx systems, the RMSD of both the pocket and the ligand, and the distance between the centers of mass of E6 and the ligand were calculated. The pocket volume was measured using POVME [38], and the protein-ligand atom contacts (*<* 3.0 Å) were evaluated using the *cpptraj* module of Amber16. The HBond plugin of VMD was employed to identify hydrogen bond events during the entire trajectory. The Δ*H*_bind_ was estimated using MM/PB(GB)SA approaches following a single trajectory protocol, and for the MM/GBSA Δ*H*_bind_ calculation, an energy decomposition per residue was performed. However, in the [E6-lig]-hx systems we estimated the Δ*H*_bind_ between the E6-lig complex and the *LxxLL* motif, i.e., E6-lig was assumed as the receptor, while the helix was assumed as the ligand.

## Results and discussion

### Compound library and ADME filtering

Small-molecule druggability of E6 protein was initially proposed by Baleja and colleagues [15]. Since then, several studies have reinforced this hypothesis, showing the effectiveness of small-molecules against E6 by *in vitro* assays [16, 17, 48]. Even some have reached clinical trials, such as Arteminol-R (an artemisinin derivative) [49], curcumin [50], or (-)-epigallocatechin-3-gallate [51]. However, none of these molecules have shown the expected effectiveness. Accordingly, to address the identification of new molecules capable of tightly binding to E6, but also with drug-likeness properties, we carried out an *in silico* analysis starting from the identification of 26 molecules (listed in S1 Table) that have previously shown to bear anti-HPV activity. Subsequently, we obtained a compound library comprised of 34,804 molecules, selected from the Zinc15 database according to their structural similarity with any of the reference compounds. The size of the library agreed with the significant structural diversity of the 26 initial molecules (S1 Fig).

The pharmacokinetic properties of the whole library were predicted and used to filter out the compounds with unsuitable ADME properties before the docking assays. In this scenario, characteristics like drug-likeness and bioavailability were prioritized at the onset expecting that, among the library screened, there would be compounds with high affinity to the target [52]. Five QikProp descriptors were used as sequential filters, allowing one violation per descriptor in order to avoid artificial distinctions between similar compounds [53]. The Lipinski’s rule of five was used for drug-likeness prediction according to the following properties: molecular mass less than 500 Da, up to 5 hydrogen bond donors, no more than 10 hydrogen bond acceptors and an octanol-water partition coefficient (*logP*_*o/w*_) no higher than 5 [24]. The Jorgensen’s rule of three was used to assess the bioavailability of each compound by estimating its solubility, permeability, and liver first-pass metabolism through the following rules: predicted aqueous solubility (*logS*_*wat*_) higher than -5.7 (with *S* in mol/dm^3^), predicted apparent Caco-2 cell rate permeability (*BIPcaco-2*) high than 22 nm/s, and number of primary metabolites up to 7 [25]. The predicted qualitative human oral absorption (2 = medium and 3 = high) and the predicted skin permeability (*logK*_*p*_ values between -8.0 y 1.0) [26] were also considered. Finally, the ADME-compliance score drug-likeness parameter (*#stars*) was used to assess the pharmacokinetic profiles of the compounds, evaluating 25 different properties within the acceptable range for 95% of known drugs [23]. S2 Table lists the criteria used for each descriptor and the number of compounds discarded. As a result, 19,119 compounds showed ADME properties in acceptable range and were selected for their evaluation during the SBVS stage.

### Molecular dynamics simulations of apo-E6 protein

**Fig 1:**
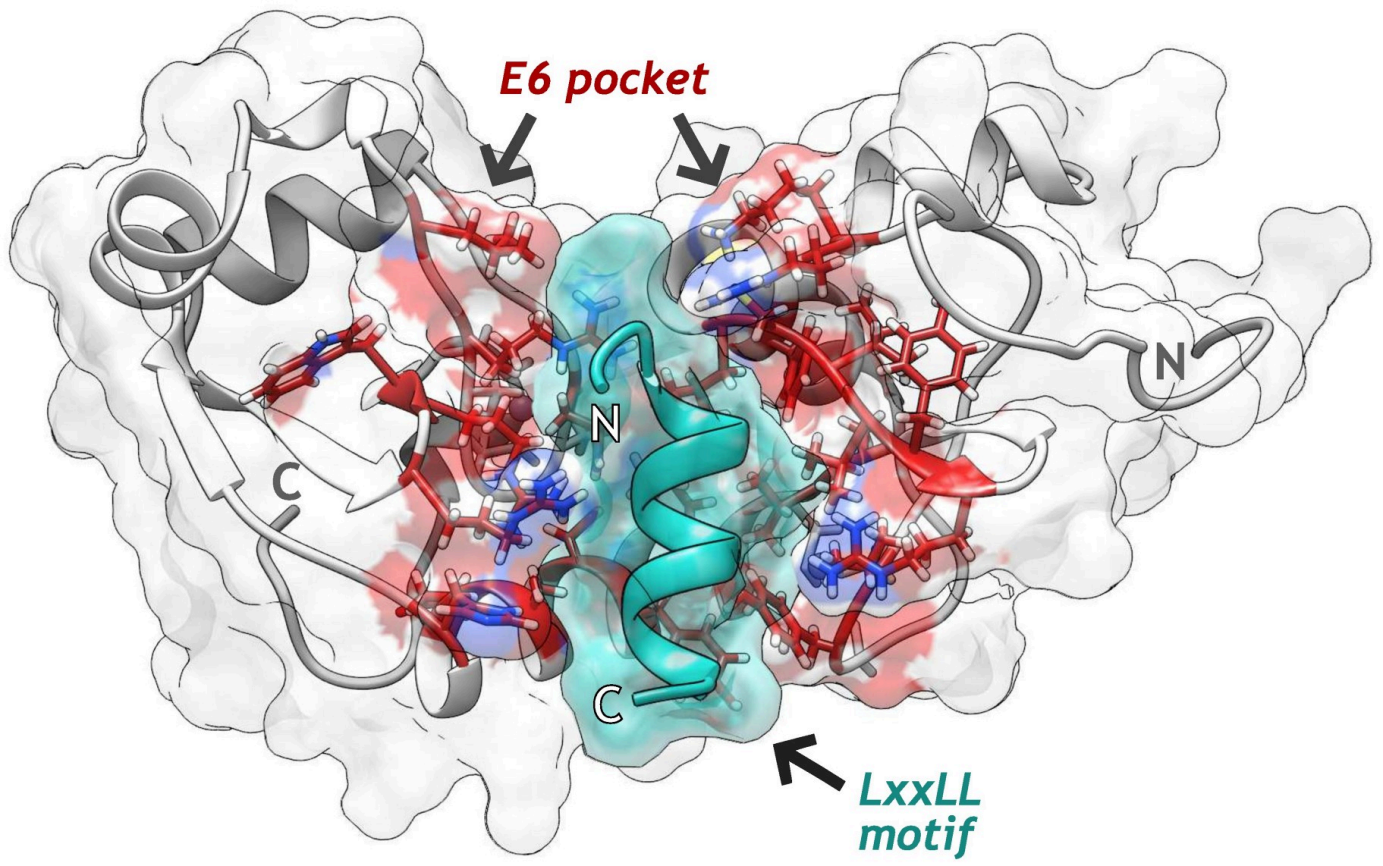
Structure of the HPV-16 E6 protein homology model in interaction with *LxxLL* helical motif (green). Residues belonging to the E6 pocket are shown in stick representation (red).

Both experimental [54, 55] and computational studies [56, 57] have documented that E6 is a very flexible protein. Since flexibility plays an important role in protein-ligand recognition [58], we decide to take into account for molecular docking calculations through an Ensemble-based docking approach. To achieve this, Modeller software v9.15 [28] was used to generate the full-length structure of E6 through homology modeling (zDOPE -0.55, RMSD of 0.8 Å) and for the loop refinement of the first seven residues missed in the template (Fig 1 and S2 Fig). Then, three independent MD assays of apo-E6 protein were carried out to obtain a set of conformers that depict the structural variability of the unbound state of E6 under physiological conditions. To compare these results, we also performed three MD assays of E6 bound to the *LxxLL* motif (E6-hx systems).

**Fig 2:**
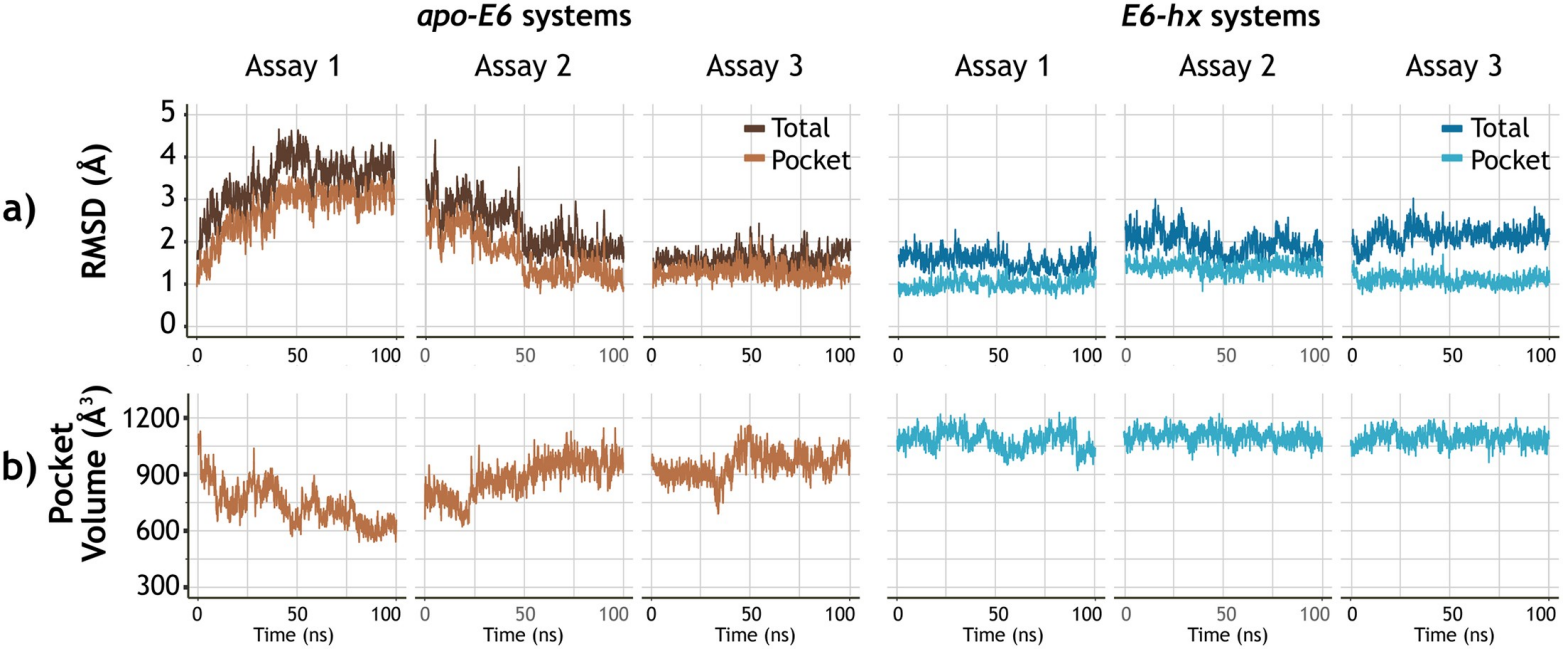
Root mean square deviation (RMSD) and pocket volume analysis of apo-E6 and E6-hx systems. **a)** RMSD analysis was carried out for the molecular dynamics simulations of each system considering both the C*α* atoms of the whole protein (*whole*) and only those belonging to the pocket (*Pocket*). Pearson correlation results between *whole* and *Pocket* RMDS values were as follows. apo-E6: A1 = 0.98, A2 = 0.96, A3 = 0.88; E6-hx: A1 = 0.54, A2 = 0.56, A3 = 0.66. **b)** E6 pocket volumes throughout MD trajectories.

The RMSD of the protein was measured over the course of the 100 ns of each trajectory. Fig 2a shows the RMSD plots of apo-E6 and E6-hx systems, respectively, considering both the residues of the entire protein and only those belonging to the pocket (more detailed figures are shown in S3 and S4 Figs). In addition, the RMSF values of each E6 residue in both systems are shown in S5 Fig. With respect to E6-hx systems, the apo state of E6 showed prominent conformational changes throughout trajectories. Also apo-E6 presented higher RMSF values, particularly in the N-terminal domain, which matches with the experimental evidence that points to the high flexibility of this region [55]. Whereas, the protein backbone remained more stable when interacted with the *LxxLL* motif. Similar behavior was reported by Shah *et al*. [56] when simulating the E6 interaction whit the *LxxLL* motifs of E6AP and IRF3. Finally, in the apo-E6 there was a high correlation (> 0.85) between the RMSD values of the pocket and those of the whole protein. This correlation suggests that the observed changes of the conformational macrostates of the unbound state of E6 mostly depended on the pocket conformation. Moreover, the pocket volume over the course of the trajectory seems to follow the conformational transition of the protein (Fig 2b). Especially in the apo-E6 systems, in which the pocket sizes were smaller.

### Conformational sampling of E6 protein

**Fig 3:**
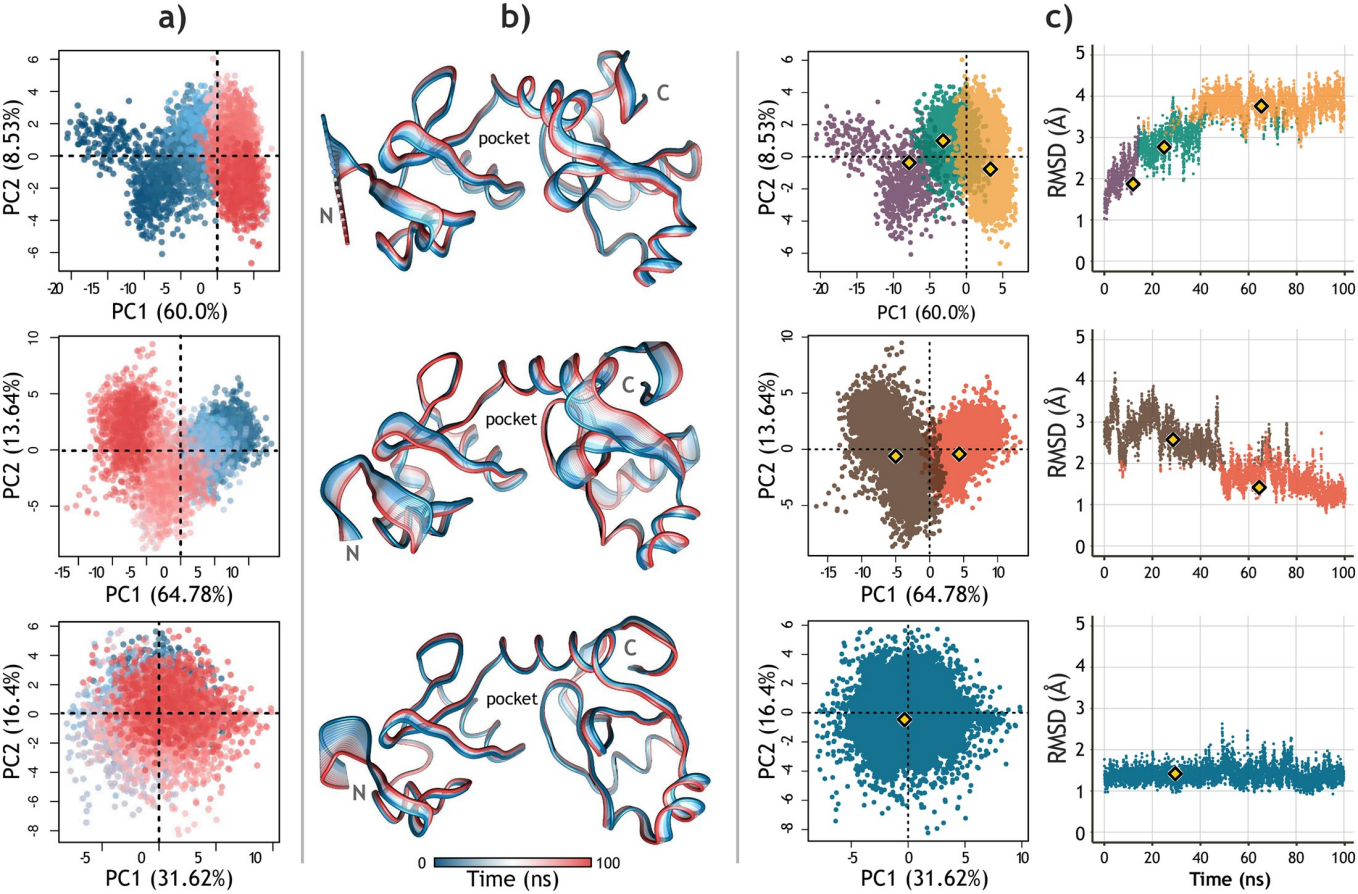
Principal Component Analysis of the three apo-E6 trajectories. **a)** Projection of the conformational distribution onto the subspace defined by the first two PCs of each assay (red: 0 ns to blue: 100 ns). **b)** Superimposed structures representing a trajectory obtained from the variance contribution of each residue in the first CP of each assay. The opening of the pocket is exhibited by the flexibility of the linker-helix joining the two Zn^2+^ binding domains. **c)** Conformational clusters of each apo-E6 trajectory displayed on the PCA scatter plot (left) and the RMSD plot (right) of each assay. Each dot represents an E6 conformer, and the yellow markers indicate the representative conformations (medoids) of each cluster.

Ensemble docking requires a set of protein conformations relevant to binding, these conformations can be obtained from MD trajectories. However, defining a protocol for selecting MD snapshots is a challenging task [59]. Here, we choose to combine the use of PCA, to reveal the dominant modes of motion of the apo-E6 protein, and geometric clustering of the binding pocket to select a reduced set of representative conformations [60, 61]. Fig 3 shows the PCA and clustering results corresponding to each of three apo-E6 systems. The cumulative contribution of principal components (PCs) in total variance is shown in S6 Fig. The first three PCs of each assay cover 75.4%, 80.7%, and 60.6%, respectively. Fig 3a shows the projection of apo-E6 conformations in the subspace spanned by the first two PCs. Also, the motion modes of the protein explained by the first PC of each trajectory are represented in Fig 3b. These structural representations emphasize the independent mobility of N-terminal and C-terminal domains, which denotes the opening and closing capacity of the pocket. The results are in accordance with previous *in silico* studies. Shah *et al*. [56] observed an “open state” of E6, pointing out the flexibility of the linker-helix joining the two Zn^2+^ binding domains. Rietz *et al*. [57] highlighted the pocket plasticity, mainly due to the movement of the side chains of the arginine residues.

Hierarchical clustering was performed considering only the data contained within the first three PCs of each apo-E6 assay. We resolved to select only a few E6 conformations because previous studies have suggested that small ensemble sizes optimize Ensemble-based docking results [58, 59, 61]. The number of clusters was decided based on the visual inspection of the RMSD plots and the dendograms generated by the hierarchical clustering itself (S7 Fig). Fig 3c presents the clusters obtained for each trajectory and the position of the representative structure of each cluster; the medoid conformation. The superposition of these six conformations, their RMDS values and their pocket volumes are shown in S8 Fig.

### Structure-based virtual screening

#### Ensemble-based virtual screening with Autodock Vina

**Fig 4:**
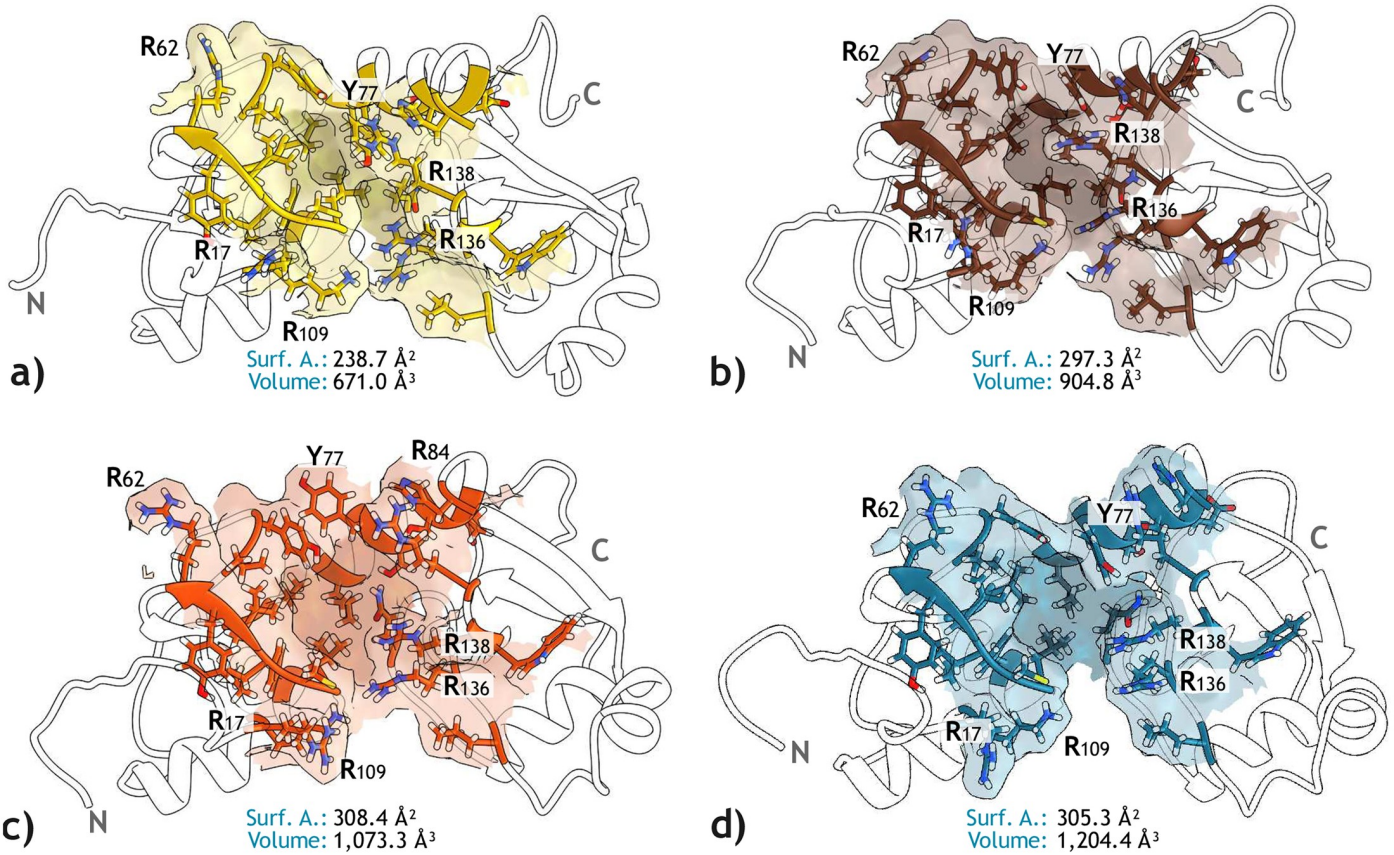
*LxxLL* binding pocket of the four E6 conformations selected for the EBD with Vina. The residues structuring the pocket of each E6 conformer and the surface representation of their side chains are shown, highlighting the position of arginine residues. The volume and surface area values are also presented. Molecular graphics were produced by using UCSF Chimera software [62].

Unlike cellular proteins that interact with cellular acidic *LxxLL* motifs through shallow surfaces, the E6 pocket consists of an original groove able to capture the *LxxLL* motif [11]. This particularity represents an opportunity for Structure-Based drug discovery against HPV. However, as explained earlier, take into account target flexibility is pivotal for optimal results. Then, an Ensemble-based docking approach was performed using four apo-E6 conformations, which represent the most populated clusters obtained in the previous step. As shown in Fig 4, these structures represent the open and closed states of E6 protein, both defined by the backbone and side chains of the pocket.

For each of the four conformations, the 19,119 compounds screened by they ADME properties were docked using Vina. Then, a set of the 3,000 top-ranked ligands was obtained. Finally, the ligands intersected in all four sets were identified and selected for further evaluation. Thus, due to the observed flexibility of the unbound state of E6, this evaluation attempted to reduce the number of false positives, assuming that the true binder molecules are more likely to interact with E6 despite the pocket plasticity.

**Fig 5:**
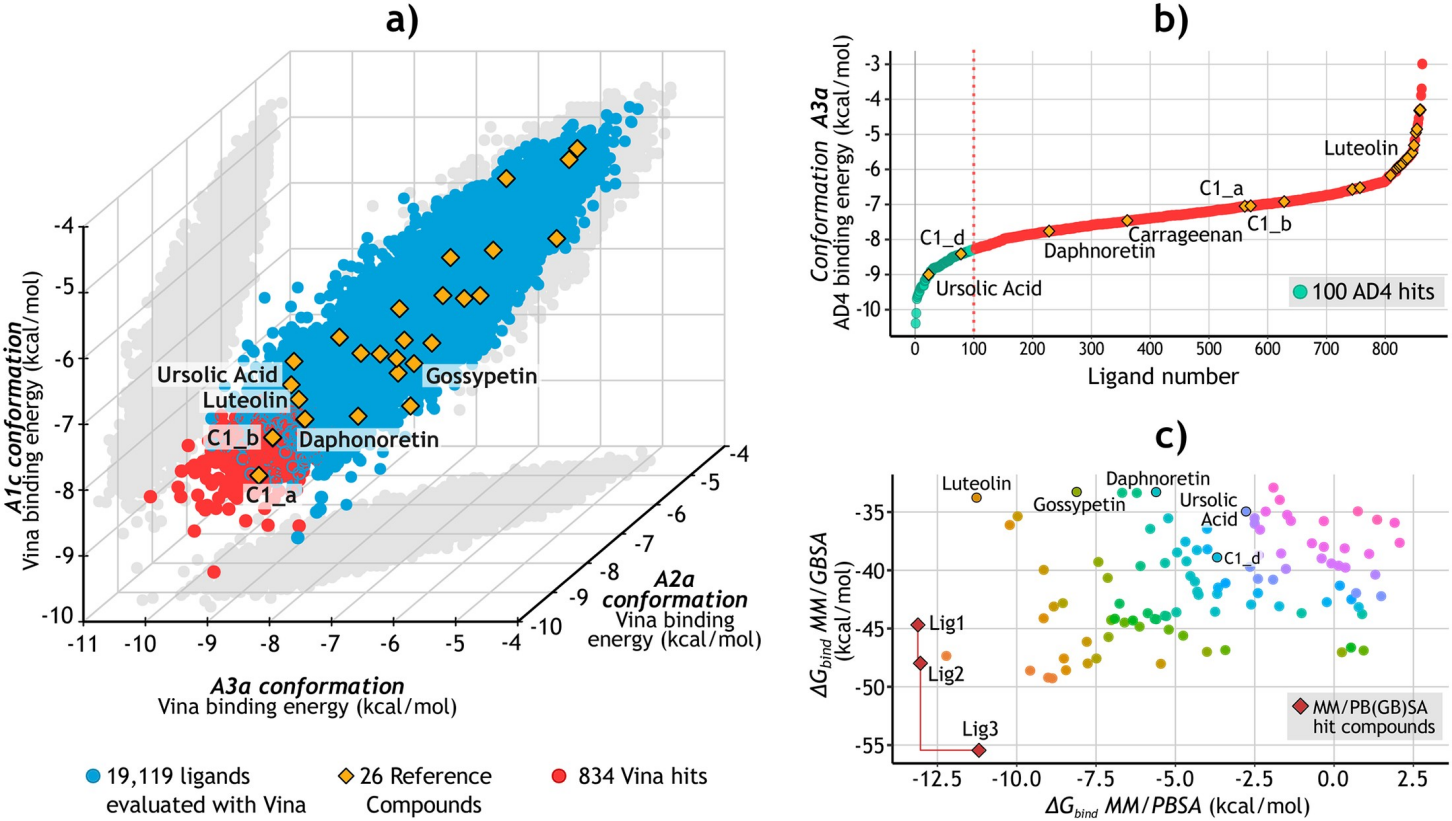
Structure-Based virtual screening. **a)** Results of the Ensemble-Based Docking using Vina. The 834 molecules with the most favorable binding values (red) were selected to be evaluated with AD4. For visualization purposes, only the results of three of the conformations (A1c, A2a, and A3a) are shown. Full results can be consulted in S9 Fig. **b)** Results of the evaluation of the 834 molecules docked to the A3a E6 conformation using AD4. The 100 ligands (green) with the lowest binding values were selected. The reference compounds were also analyzed. **c)** Rescoring of the AD4 top-ranked ligands using MM/PB(GB)SA. The Pareto front is defined by the red line connecting the three optimal solutions, corresponding to the three final candidate compounds: Lig1 (ZINC111606147), Lig2 (ZINC362643639), and Lig3 (ZINC96096545).

Part of the results is presented in Fig 5a, where a 3D scatter plot represents the distribution of the ligands according to their docking score for three of the four E6 conformations. The red dots at the bottom left of the graph correspond to those ligands intersected in the top-ranked sets, with a total of 834 compounds. Interestingly, the A3a conformation showed the lowest Vina scores while the A1c conformation had the highest (S9 Fig). This result seems to be related to the different pocket sizes, where the tightest conformation had the least favorable binding affinities. Finally, the Spearman’s ranking correlation coefficients between each pair of conformations are presented in S3 Table, showing *ρ* values greater than 0.72.

### Docking simulations with Autodock 4

For the second docking stage, AD4 was used to dock the 834 ligands to only one apo-E6 conformation (Fig 4d). Results are plotted in Fig 5b, which highlights the 100 top-ranked ligands for this process, showing values between -8.5 and -10.5 kcal/mol. It also shows the results for the 26 reference compounds, which were evaluated even if they were discarded in the previous stage. Additionally, S10a Fig) presents a heat map of Tanimoto coefficients comparing these 100 ligands and the 26 reference compounds. S10b Fig) indicates which pocket residues of E6 have contributed the most to the formation of hydrogen bonds by interacting with these 100 ligands.

### Binding free energy calculations of docked complexes

MM/GBSA and MM/PBSA methods have proved to be more accurate than the scoring functions used in docking evaluations, and are faster than more rigorous approaches such as free energy perturbation and thermodynamic integration [43]. MM/PB(GB)SA were employed to rescore the docking poses of the 100 ligands selected from the AD4 assay. Nevertheless, the results showed no correlation (*ρ* = 0.35) between Δ*H*_bind_ values of both analyses. This discrepancy between Δ*H*_bind_ estimations of the two methods has been pointed out previously, indicating that the success or failure of either of the two depends on the studied system [63]. However, to the best of our knowledge, there are no experimental binding affinities reported for the selected reference compounds to compare with, and to determine which approach has the more accurate results. For this reason, we resolved to take into account the two methods for the selection of the candidate molecules, assuming that the true E6 binders will show favorable Δ*H*_bind_ values in both of them. Thus, using a Pareto ranking method [47] the three ligands comprising the non-dominated frontier were selected as the final candidate compounds to be evaluated by MD (Fig 5c). The Zinc15 identifiers of luteolin and the three candidates (hereinafter referred to as Lig1, Lig2, and Lig3) are shown in Table 1. The structural characteristics of the compounds and their predicted values regarding drug-likeness and bioavailability are also presented. Only Lig1 and Lig3 fully satisfy the Lipinski’s and Jorgensen’s rules criteria. However, despite Lig2 has a *logS*_*wat*_ below the rule’s threshold, its value still falls within the range for 95% of known drugs [23].

**Table 1:** Screened candidate compounds and their pharmacokinetic properties.

Ligand name	Luteolin	Lig1	Lig2	Lig3
**Zinc15 ID**	ZINC18185774	ZINC111606147	ZINC362643639	ZINC96096545
**MW (g/mol)**	286.2	319.3	337.4	396.8
**Number of rings**	3	4	4	3
**Heavy atoms**	21	24	24	28
**Rotatable bonds**	1	3	4	4
**H-bond donors**	4	3	3	3
**H-bond acceptors**	6	5	4	5
^a^***logP***_*o/w*_	0.93	2.28	3.69	3.18
^b^ ***logS*** *wat*	-3.03	-4.42	-5.94	-5.54
^c^***BIPcaco-2*** **(nm/s)**	40.80	230.66	494.27	290.28
^d^**Number of metabolites**	4	3	3	2

^a^ Predicted logarithm of partitioning coefficient between octanol and water phases (range for 95% of drugs [23]: -2.0 to 6.0).

^*b*^ Predicted logarithm solubility, with *S* in mol/dm^3^ (range for 95% of drugs [23]: -6.0 to 0.5).

^*c*^ Predicted apparent Caco-2 cell rate permeability in nm/s (range for 95% of drugs [23]: *<* 5 low, *>* 100 high).

^*d*^ Number of likely metabolic reactions during liver first-pass metabolism (range for 95% of drugs [23]: 1 to 8).

**Fig 6:**
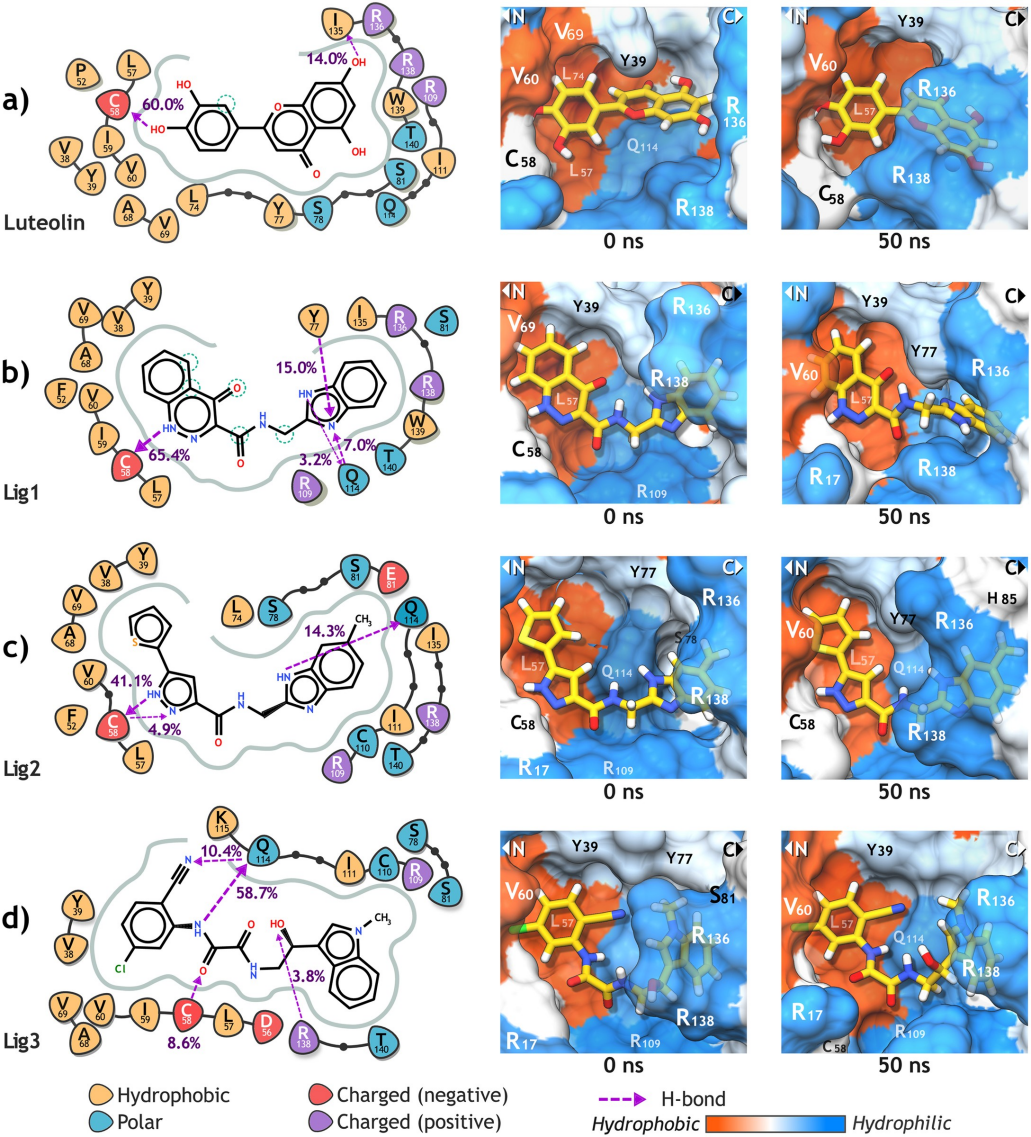
Ligand interaction diagrams of luteolin (a) and the three candidate compounds (b, c, d) with the E6 pocket residues. For each ligand, the involving hydrogen bonds and its occupancy are highlighted. Additionally, the interaction structures at the beginning (0 ns) and the end (50 ns) of the MD production are presented. As shown in 2D diagrams, C58 was predicted as a deprotonated cysteine by PROPKA.

We decided to take luteolin as a positive control due to its identification as a potential inhibitor of the E6-E6AP interaction by Cherry *et al*. [16]. Its anti-HPV activity was proved in CaSki, HeLa, and SiHa cell lines, rising p53 protein levels and decreasing the cell viability [16, 64]. Furthermore, the direct E6-luteolin binding was confirmed by *in vitro* assays, and docking simulations suggested that this interaction occurs through the E6 pocket [16]. Although herein luteolin had moderate binding scores during the SBVS stage, its docked pose agreed with the one obtained by Cherry *et al*. [16] (Fig 6a), and its evaluation with MM/PBSA indicated favorable relative Δ*H*_bind_ values (Fig 5c).

### Evaluation of best three candidate compounds by MD

MD was employed for the postprocessing of the E6-lig complexes to validate and refine the docking solutions [65] of the Lig1, Lig2, and Lig3 molecules. In this analysis, we aimed (i) to evaluate the stability and Δ*H*_bind_ of the E6-ligand complex, and (ii) to estimate the effect of the docked ligand over the E6 binding affinity to the *LxxLL* motif (hx) of E6AP. For this purpose, the docking pose of each compound was used to simulate 50 ns of two separate complexes; the E6-lig and [E6+lig]-hx systems.

### Trajectory analysis and Δ*H*bind of E6-lig systems

The trajectory analysis results and Δ*H*_bind_ estimations of each E6-lig system are summarized in Table 2. Fig 6 shows the interaction maps with E6 pocket of luteolin, Lig1, Lig2 and Lig3. The four compounds had low RMSD average when compared with their docked pose, indicating a high degree of stability over the course of the trajectory, even after the SA stage. Particularly, Lig2, and Lig3 had the lowest values, keeping their initial pose after 50 ns (Fig 6c and 6d). Likewise, the E6 pocket volume remained near to its initial values (Fig 4d); except the E6-luteolin complex in which the pocket showed a semi-closed conformation. Additionally, the RMSF values of the pocket and the whole protein were lower when docked with Lig2 and Lig3. These values are between those of the apo-E6 systems (1.4 *±* 0.3) and E6-hx systems (0.7 *±* 0.1) (S10 Fig).

**Table 2:** Trajectory analysis for each of the E6-lig systems along 50 ns of MD.

Ligand name/ Zinc15 ID	RMSD ligand (Å)	RMSD protein (Å)	Dis- tance E6-lig (Å)	RMSF of pocket (Å)	E6 pocket volume (Å^3^)	ΔΔ*H*^*GB*^ (kcal/ mol)	ΔΔ*H*^*P B*^ (kcal/ mol)
Luteolin	0.9 *±*0.2	3.8 *±*0.4	5.2 *±*0.5^a^	1.0 *±* 0.6	1,006 *±* 92	0.0 *±* 4.1	0.0 *±* 4.8
Lig1	1.1 *±*0.6	4.2 *±*0.5	5.0 *±*0.7^a^	0.9 *±* 0.4	1,214 *±*110	1.3 *±* 3.6	3.0 *±* 5.5
Lig2	0.7 *±*0.2	3.4 *±*0.4	4.5 *±*0.4^a^	0.8 *±* 0.3	1,164 *±*114	-5.3 *±*3.3	**-0.4** *±*4.7*
Lig3	0.7 *±*0.2	3.4 *±*0.3	4.0 *±*0.4^a^	0.8 *±* 0.5	1,194 *±*149	**-8.2** *±*3.8*	1.0 *±* 4.5

* The best results according to the evaluated objective. The highest ΔΔ*H*_*bind*_ values are highlighted.

**a** Distance between E6 protein and the docked ligand in *E6-lig* systems.

The protein-ligand interaction analysis showed that the backbone of the residue C58 had a high occupancy of the H-bonds observed for the four ligands. Rietz *et al*. [57] reported a similar behavior when evaluated some flavon analogs and benzopyran derivatives. Nevertheless, we also found Q114 playing a significant role in protein-ligand interactions, forming H-bonds with the three candidate compounds. Moreover, the residue-based free energy decomposition analysis indicated that Q114 contributed the most to the total Δ*H*_bind_ to Lig2 and Lig3, while C58 was the major contributor to the Δ*H*_bind_ with Lig1 and luteolin (S12 Fig).

Residues K18, L57, and R109 also had favorable affinities with the three candidates, showing favorable electrostatic interaction energies (Δ*H*_ele_) and van der Waals interaction energies (Δ*H*_vdW_). Interestingly, these residues along with Q114, are highly conserved among HR HPVs. Instead, luteolin formed an H-bond with I135 at the end of the simulation, just after the displacement of the E6 C-terminal domain, closing the pocket and allowing luteolin to binding to the carbonyl oxygens of C58 and I135 at the same time (Fig 6a and S12 Fig).

Throughout the E6-Lig1 system simulation, the oxocinnoline group of Lig1 remained stable over the hydrophobic pocket surface, keeping close atom-pair contacts with V38 and L57. On the contrary, after the first 28 ns, the benzimidazole group flipped from its initial position promoting the formation of H-bonds with the hydroxyl group of Y77, stabilizing the ligand during the last 20 ns (Fig 6b and S12 Fig). In the case of Lig2, it kept close contacts with Y39, V69, and L57 through its thiophene and pyrazole rings, respectively. Also, it showed favorable Δ*H*_ele_ values interacting with R17, K18, R109, and Q114 through its carboxamide group (Fig 6c and S11 Fig). For E6-Lig3 system, the 5-chloro-2-cyanophenyl ring of Lig3 interacts with the hydrophobic region of the pocket, maintaining close contacts with L57 and V69. Q114 established favorable electrostatic and van der Waals interactions with oxamide group atoms, as did K18 and R109 (Fig 6d and S11 Fig).

Finally, when compared with luteolin, the compounds Lig2 and Lig3 displayed lower average ΔΔ*H*_bind_ values calculated by MM/GBSA. However, for MM/PBSA the average ΔΔ*H*_bind_ values were similar among luteolin, Lig2, and Lig3; and slightly more unfavorable for Lig1. These are very promising results, especially when considering the assumption that a correct docking pose displays stable and specific interactions with the target through the trajectory [65], and that binding of a high-affinity ligand stabilizes the protein into a preferred state, reducing the conformational entropy of the complex [60].

### Candidate compounds decrease E6-*LxxLL* binding affinity

Each [E6+lig]-hx system consisted of the *LxxLL* motif placed inside the E6 pocket of an E6-ligand complex, but avoiding the overlapping with the atoms of the ligand. Thereby we aimed to test both the stability of the E6-ligand complex in the presence of the *LxxLL* motif and the effect of the ligand on the E6-E6AP interaction. The relative Δ*H*_bind_ calculations were performed to evaluate the affinity of each E6-ligand complex ([E6-lig]) to *LxxLL* (hx), having as a reference an [E6]-hx system. The results of the MD are presented in Table 3, and are described in more detail below.

**Table 3:** Trajectory analysis results for each of [E6+lig]-hx systems during 50 ns of MD.

Ligand name/ system	RMSD ligand (Å)	RMSD protein (Å)	Dis- tance E6-hx (Å)	RMSF of pocket (Å)	E6 pocket volume (Å^3^)	ΔΔ*H*^*GB*^ (kcal/ mol)	ΔΔ*H*^*P B*^ (kcal/ mol)
*LxxLL* motif *[E6]-hx*	NA	3.4 *±* 0.3	9.6 *±*0.7^a^	1.0 *±* 0.7	1,301 *±* 54	0.0 *±* 9.5	0.0 *±*13.7
Luteolin *[E6+luteolin]-hx*	0.8 *±*0.1	6.1 *±* 0.5	14.1 *±*0.5^a^	1.0 *±* 0.6	1,098 *±* 78	20.6 *±*8.1	-9.6 *±*9.5
Lig1 *[E6+Lig1]-hx*	1.3 *±*0.5	4.2 *±* 0.5	13.6 *±*0.4^a^	0.9 *±* 0.6	1,279 *±* 65	**31.2** *±*6.0*	4.6 *±* 7.4
Lig2 *[E6+Lig2]-hx*	0.7 *±*0.1	3.9 *±* 0.3	13.2 *±*0.4^a^	1.0 *±* 0.5	1,360 *±*155	30.8 *±*7.3	**6.9** *±*9.5*
Lig3 *[E6+Lig3]-hx*	2.0 *±*0.5	4.5 *±* 0.3	13.7 *±*0.5^a^	0.9 *±* 0.8	1,302 *±* 62	17.3 *±*5.9	2.8 *±* 8.3

* The best results according to the evaluated objective. The highest ΔΔ*H*_*bind*_ values are highlighted.

**a** Distance between E6 protein and *LxxLL* helical motif in *[E6+lig]-hx* systems.

In general terms, the E6-ligand interactions were similar to those observed in the E6-lig systems. All ligands preserved its docking pose, except for Lig3 that after 10 ns of MD turned its indole group due to a NH-*π* interaction with R138. In the case of [E6+luteolin]-hx, the E6 protein showed high RMSD values and similar pocket volumes to the E6-luteolin system, denoting a semi-closed state of the E6 protein and high fluctuations of the N-terminal residues (S11 Fig). For the systems involving Lig2 and Lig3, their E6 pocket volumes were larger than their E6-lig systems. This was mainly because the position of R136 side chain that in E6-lig systems was closed to the ligand, but in the [E6+lig]-hx systems the helix prevented this approximation.

The E6-ligand interaction analysis showed the same H-bonds formation involving the C3 hydroxyl of luteolin and the C58 residue (85.2% occupancy), but this time the H-bonds with I135 was not observed. Instead, luteolin established and H-bonds with the residue Q164 (51.0% occupancy) belonging to the helical motif. Lig1 showed H-bonds again with C58 (72.6% occupancy) and Q114 (8.0% occupancy), but not with Y77 due to its benzimidazole group kept its docked position. Lig2 and Lig3 also showed the same H-bonds formations with C58 (Lig2: 58.2% occupancy, Lig3: 33.2% occupancy) and Q114 (Lig2: 8.1% occupancy, Lig3: 54.6% occupancy). Additionally, Δ*H*_bind_ decomposition per-residue indicated a slight increase in the Δ*H*_bind_ values for luteolin, Lig1, and Lig3, while for Lig2 the affinity values were similar to the E6-Lig2 system (S13 Fig).

**Fig 7:**
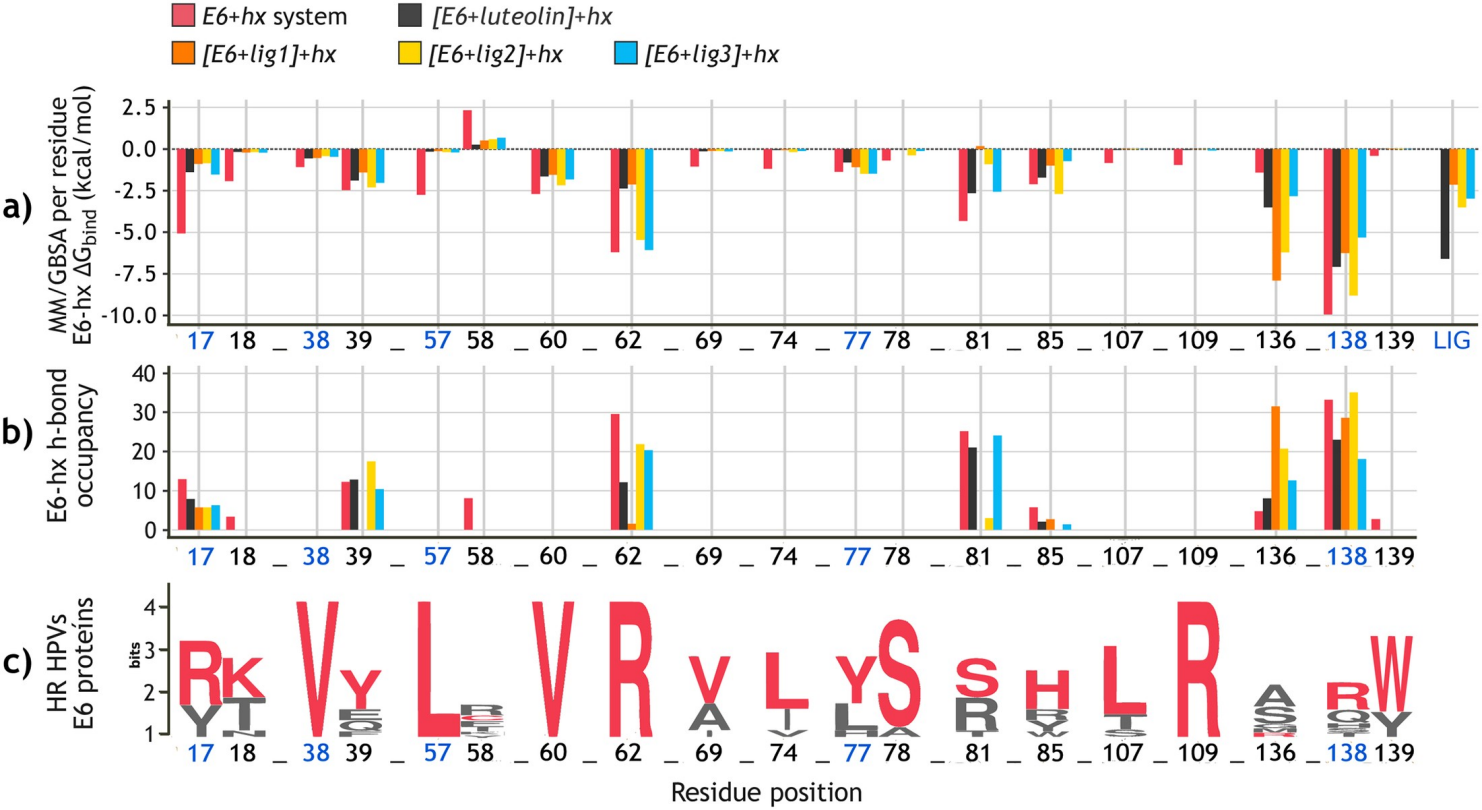
Efect of the docked compounds to E6 pocket over the E6-*LxxLL* binding affinity. **a)** MM/GBSA Δ*H*_bind_ decomposition per E6 pocket residue of each of the four [E6+lig]-hx systems and [E6]-hx system. The Δ*H*_subtotal_ (kcal/mol) values of each ligand are also presented (right). Full results can be consulted in S14 Fig. **b)** H-bonds occupancy between E6 pocket residues and *LxxLL* motif over 50 ns of MD. **c)** Sequence logo of E6 pocket of the most prevalent HR HPV types, depicting the degree of amino acid conservation at each position. Red letters indicate the corresponding residue of HPV-16 E6 protein.

Lastly, the last two columns of Table 3 show the main results for the relative Δ*H*_bind_ calculations between the E6-ligand complex and the *LxxLL* motif. As can be seen, the three candidate compounds increased the ΔΔ*H*_bind_ values when compared with [E6]-hx system, and even Lig1 and Lig2 did it better than luteolin. As expected, this implies that the presence of the ligand inside the E6 pocket decreases the E6-*LxxLL* affinity. Fig 7 details these results displaying the effect of each compound over the interaction of the *LxxLL* motif with E6 pocket residues. According to Fig 7a, the four ligands increased the MM/GBSA Δ*H*_bind_ energy contribution of residues R17, K18, L57, S81, and R138. Moreover, each docked ligand prevent the H-bonds formation between the helix and some of these residues (Fig 7b). In some cases like C58 and R136, the compounds made the E6-*LxxLL* interaction more favorable, allowing H-bonds events that were not observed in the [E6]-hx system. The lowest ΔΔ*H*_bind_ energies were for Lig3 and luteolin systems, but surprisingly, the latter had even lower Δ*H*_bind_ values than [E6]-hx system. On the other hand, Lig1 and Lig2 showed the highest ΔΔ*H*_bind_ in both MM/GBSA and MM/PBSA methods.

## Conclusions

In the present study, we conducted an *in silico* methodology combining ADME prediction, SBVS, and MD to identify new compounds able to inhibit the E6-E6AP interaction. First, 34,804 molecules were obtained from their structural similarity with 26 reference compounds that have shown some anti-HPV activity. Then, preliminary filtering was applied predicting the drug-likeness and the pharmacokinetic properties of each compound. The procedure leds to the selection of 19,119 molecules with favorable ADME profiles, followed by its evaluation through SBVS against the *LxxLL* binding pocket of the HPV-16 E6 protein. Thereby we combined homology modelling, MD, PCA, and geometric clustering to identify four E6 conformations to perform Ensemble-based docking simulations. These four structures represented the main conformational changes of the E6 pocket observed over the course of the trajectories. The results further support the idea of the E6 protein flexibility and suggest the pocket’s ability to open and close through side-chain fluctuations and backbone motions.

After the SBVS stage, the binding modes to E6 of the 100 top-ranked ligands were rescoring employing the MM/PB(GB)SA methodologies. The procedure afforded the selection of three final candidate compounds, namely Lig1 (ZINC111606147), Lig2 (ZINC362643639), and Lig3 (ZINC96096545), to be further evaluated by MD. Along the trajectories of the E6-ligand systems, the docked molecule remained in its pose and limited the protein flexibility, especially in the case of Lig2 and Lig3 compounds. Favorable interactions were established with the residues K18, L57, R109, and Q114; all of them highly conserved among the E6 proteins of HR HPVs types. When compared with the reference compound luteolin, Lig2 and Lig3 had higher affinity to the E6 protein. Lastly, the [E6+lig]-*LxxLL* system evaluation corroborated the E6-ligand complex stability despite the presence of *LxxLL*, and predicted the decrease in the E6-*LxxLL* affinity due to the docked compound.

When taken together, these results represent a new starting point for the development of anti-HPV drugs based on the disruption of E6-E6AP interactions. Since this study has only contemplated computational analysis, it is still necessary the validation of the last hit molecules by *in vitro* experiments. But beyond this, the information obtained along SBVS and MD provides new insights into the importance of the E6 protein dynamics and the leading interactions that could enhance the binding between the protein and its potential inhibitor. We suggest that further research should take these results into account in both discovery and lead optimization stages to achieve a successful development of anti-HPV drugs.

## Supporting information

S1 Fig Tanimoto plot comparing the 26 reference compounds obtained from the literature. (PDF)

S2 Fig Modelling and refinement of the full-length structure of the HPV-16 E6 protein. (PDF)

S3 Fig RMSD of the E6 protein over the course of the trajectory of the three apo-E6 systems assays. (PDF)

S4 Fig RMSD of the E6 protein over the course of the trajectory of the three E6-hx systems assays. (PDF)

S5 Fig RMSF values of the E6 protein in the apo-E6 and E6-hx systems. (PDF)

S6 Fig Principal Component Analysis of the three apo-E6 trajectories. (PDF)

S7 Fig Dendograms resulting from hierarchical clustering analysis of the molecular dynamics trajectories of the apo-E6 system. (PDF)

S8 Fig Representative conformations of E6 protein. (PDF)

S9 Fig Ensemble-based Docking results performed with Autodock Vina. (PDF)

S10 Fig Analysis of the 100 top-ranked ligands according to Autodock 4 score. (PDF)

S11 Fig RMSF values of the E6 protein in the E6-lig and [E6+lig]-hx systems. (PDF)

S12 Fig MM/GBSA binding free energy (BFE) decomposition per residue of each of the four E6-lig systems. (PDF)

S13 Fig Molecular dynamics of the protein-ligand-*LxxLL* ([E6+lig]-hx) complexes (50ns). (PDF)

S14 Fig MM/GBSA binding free energy (BFE) decomposition per residue of each of the four [E6+lig]-hx systems, evaluating E6-ligand interaction. (PDF)

S15 Fig MM/GBSA binding free energy decomposition per residue of each of the four [E6+lig]-hx systems, evaluating E6-hx interaction. (PDF)

S1 Table Twenty-six reference compounds identified from the literature. These compounds have shown activity against HPV-positive cells in *in vitro* assays, and/or against E6 protein in *in silico* approaches. References corresponding to each molecule are also included.
(PDF)

S2 Table Number of compounds filtered out for each *QikProp* property. (PDF)

S3 Table Spearman ranking correlation between the Vina ligand rankings for each pair of apo-E6 conformations. (PDF)
