# Supplementary material for: Molecular modeling simulation studies reveal new potential inhibitors against HPV E6 protein
Source: PLoS One. 2019 Mar 15;14(3):e0213028. doi: 10.1371/journal.pone.0213028 (PMC6420176; doi:10.1371/journal.pone.0213028)
Supplement: S1 Fig — (PDF) [file pone.0213028.s001.pdf]

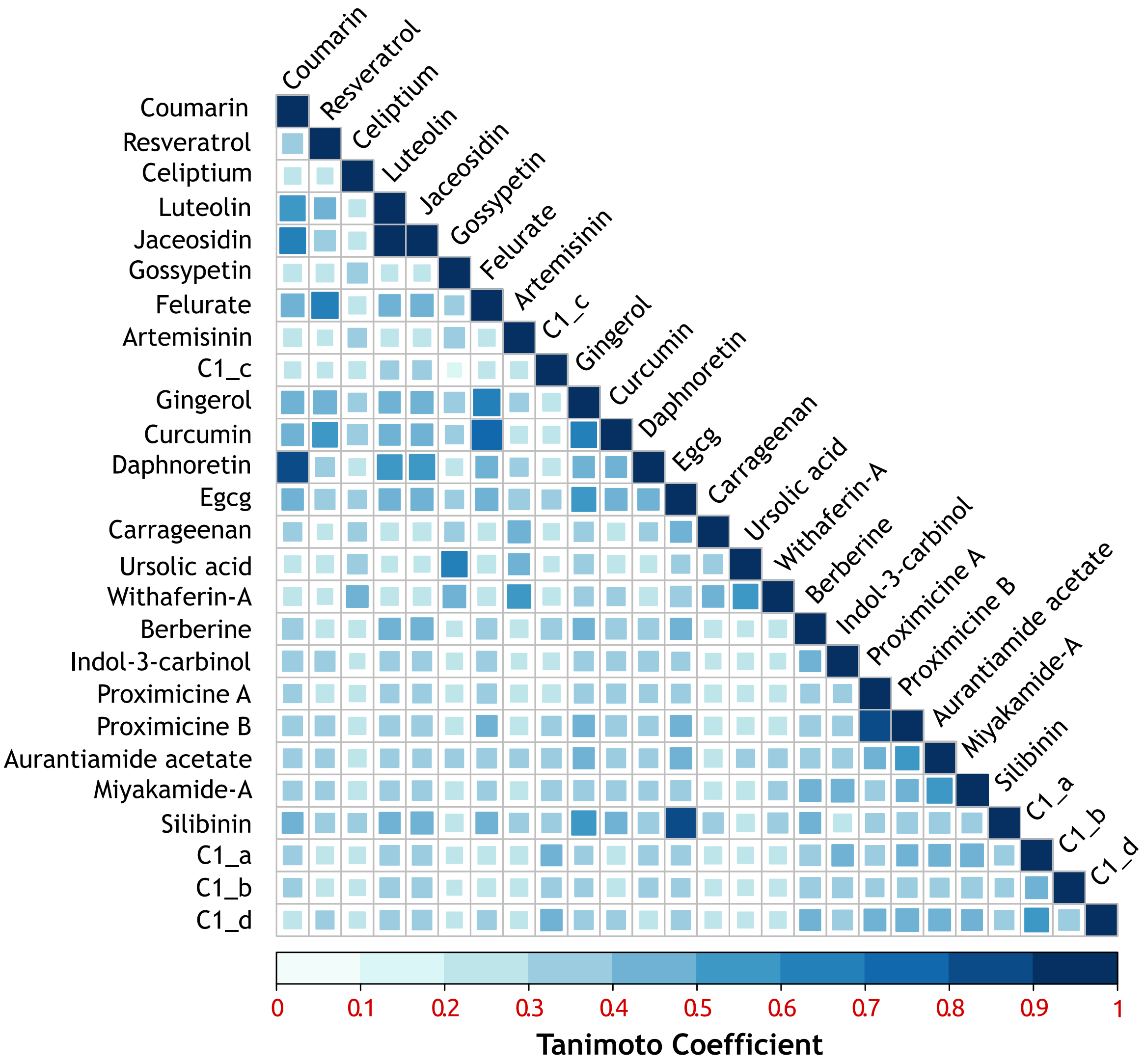

Figure S1: Tanimoto plot comparing the 26 reference compounds obtained from the literature. Tanimoto coefficients ($S_{A,B} = 2c/(a+b)$) calculated for each pair of molecules. Values close to 1 (dark blue) imply an absolute 2D structural similarity between the compared molecules.
