# Supplementary material for: Molecular modeling simulation studies reveal new potential inhibitors against HPV E6 protein
Source: PLoS One. 2019 Mar 15;14(3):e0213028. doi: 10.1371/journal.pone.0213028 (PMC6420176; doi:10.1371/journal.pone.0213028)
Supplement: S2 Fig — (PDF) [file pone.0213028.s002.pdf]

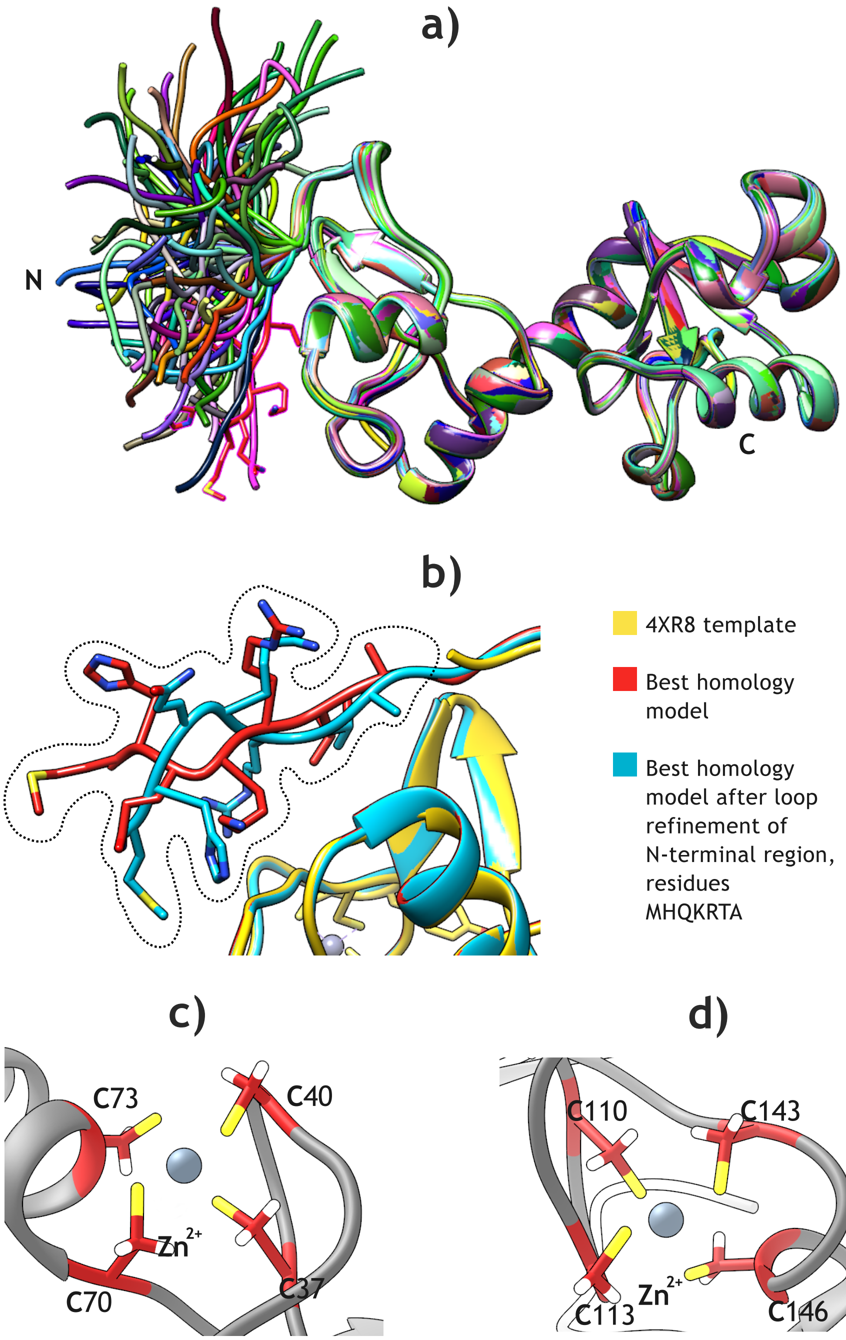

Figure S2: Modelling and refinement of the full-length structure of the HPV-16 E6 protein. a) 100 homology models generated by Modeller v9.15 using the UiniProt P03126 sequence of HPV-16 E6 protein and the crystallographic structure of HPV-16 E6 protein as the template (PDB ID: 4XR8). The missed residues of the template, corresponding to the first seven amino acids of the HPV-16 E6 protein sequence (MHQKRTA), were taken into account. **b)** Red: selected homology model of HPV-16 E6 protein (zDOPE score -0.55). Blue: modeled N-terminal region (residues MHQKRTA) after loop refinement using Modeller (blue). **c)** and **d)** The two $(Cys)_4Zn^{2+}$ fingers were modeled using the Zinc Amber Force Field (ZAFF) [31] through the tleap module of Amber Tools [32].
