# Supplementary material for: Molecular modeling simulation studies reveal new potential inhibitors against HPV E6 protein
Source: PLoS One. 2019 Mar 15;14(3):e0213028. doi: 10.1371/journal.pone.0213028 (PMC6420176; doi:10.1371/journal.pone.0213028)
Supplement: S3 Fig — (PDF) [file pone.0213028.s003.pdf]

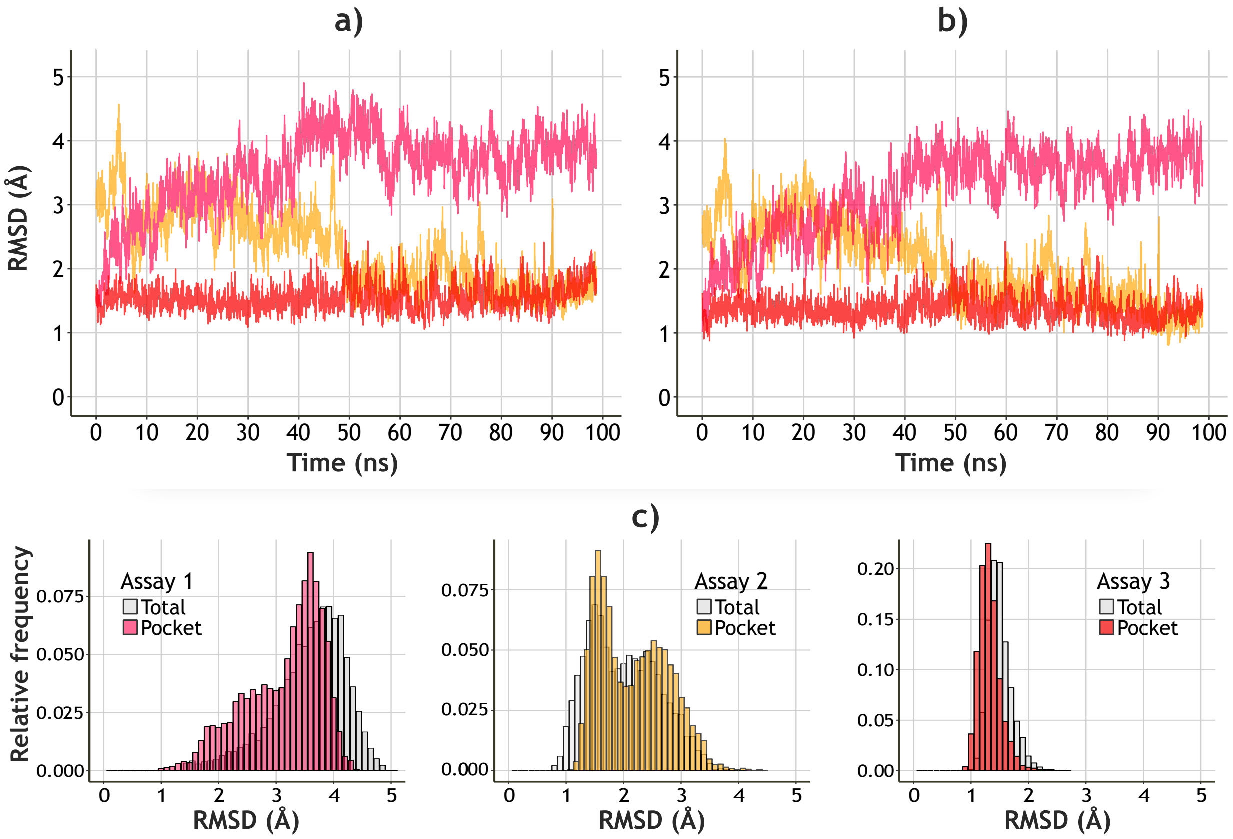

Figure S3: RMSD of the E6 protein over the course of the trajectory of the three apo-E6 systems assays. a) RMSD values for the Cα of the E6 protein in each assay. Homology model was used as reference structure. **b)** RMSD values considering only the C of the residues belonging to the E6 pocket. **c)** Histograms showing the RMSD values of the whole backbone of E6 protein (Total) and the pocket for each assay. Although small differences are observed, the conformational variation of the pocket's backbone is similar to whole E6 backbone.
