# Supplementary material for: Molecular modeling simulation studies reveal new potential inhibitors against HPV E6 protein
Source: PLoS One. 2019 Mar 15;14(3):e0213028. doi: 10.1371/journal.pone.0213028 (PMC6420176; doi:10.1371/journal.pone.0213028)
Supplement: S4 Fig — (PDF) [file pone.0213028.s004.pdf]

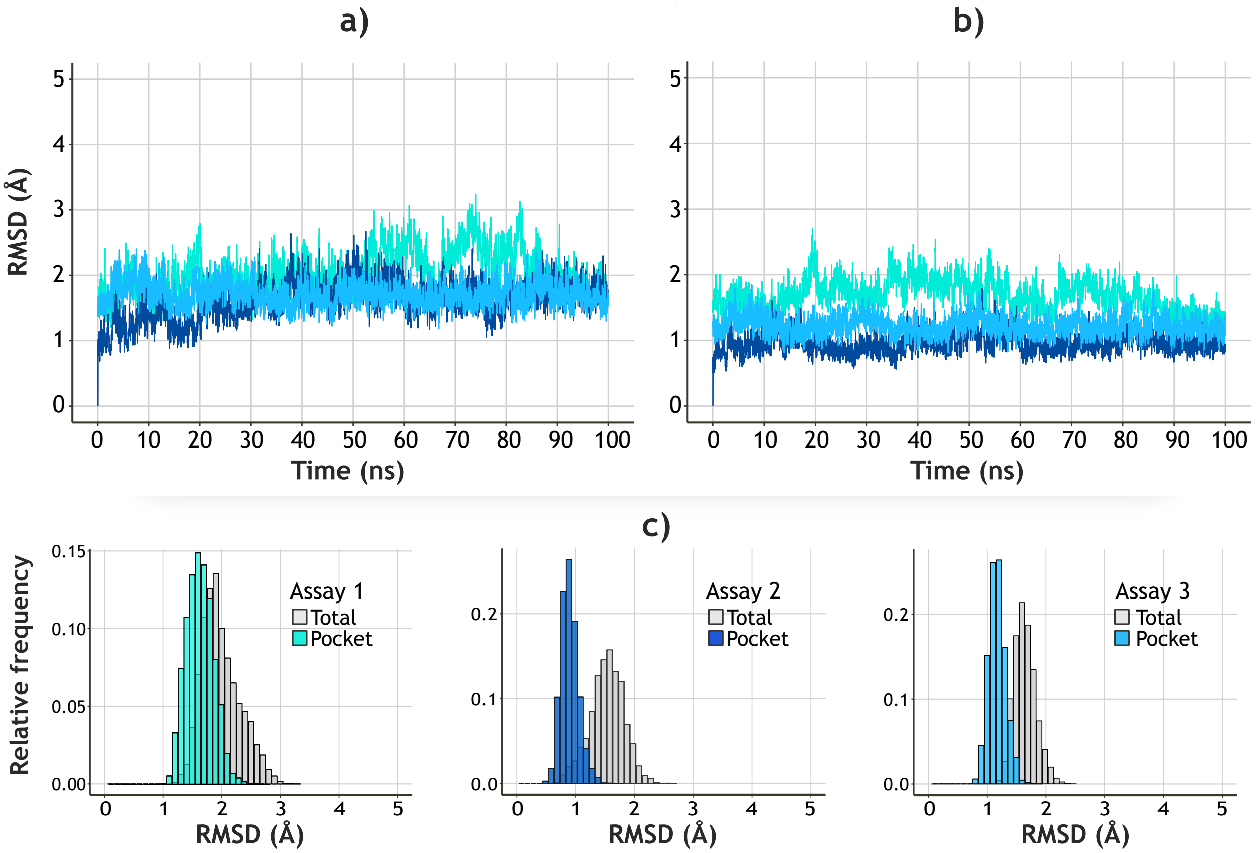

Figure S4: RMSD of the E6 protein over the course of the trajectory of the three E6-hx systems assays. **a)** RMSD values for the Cα of the E6 protein in each assay. Homology model was used as reference structure. **b)** RMSD values considering only the C of the residues belonging to the E6 pocket. **c)** Histograms showing the RMSD values of the whole backbone of E6 protein (Total) and the pocket for each assay.
