# Supplementary material for: Molecular modeling simulation studies reveal new potential inhibitors against HPV E6 protein
Source: PLoS One. 2019 Mar 15;14(3):e0213028. doi: 10.1371/journal.pone.0213028 (PMC6420176; doi:10.1371/journal.pone.0213028)
Supplement: S5 Fig — (PDF) [file pone.0213028.s005.pdf]

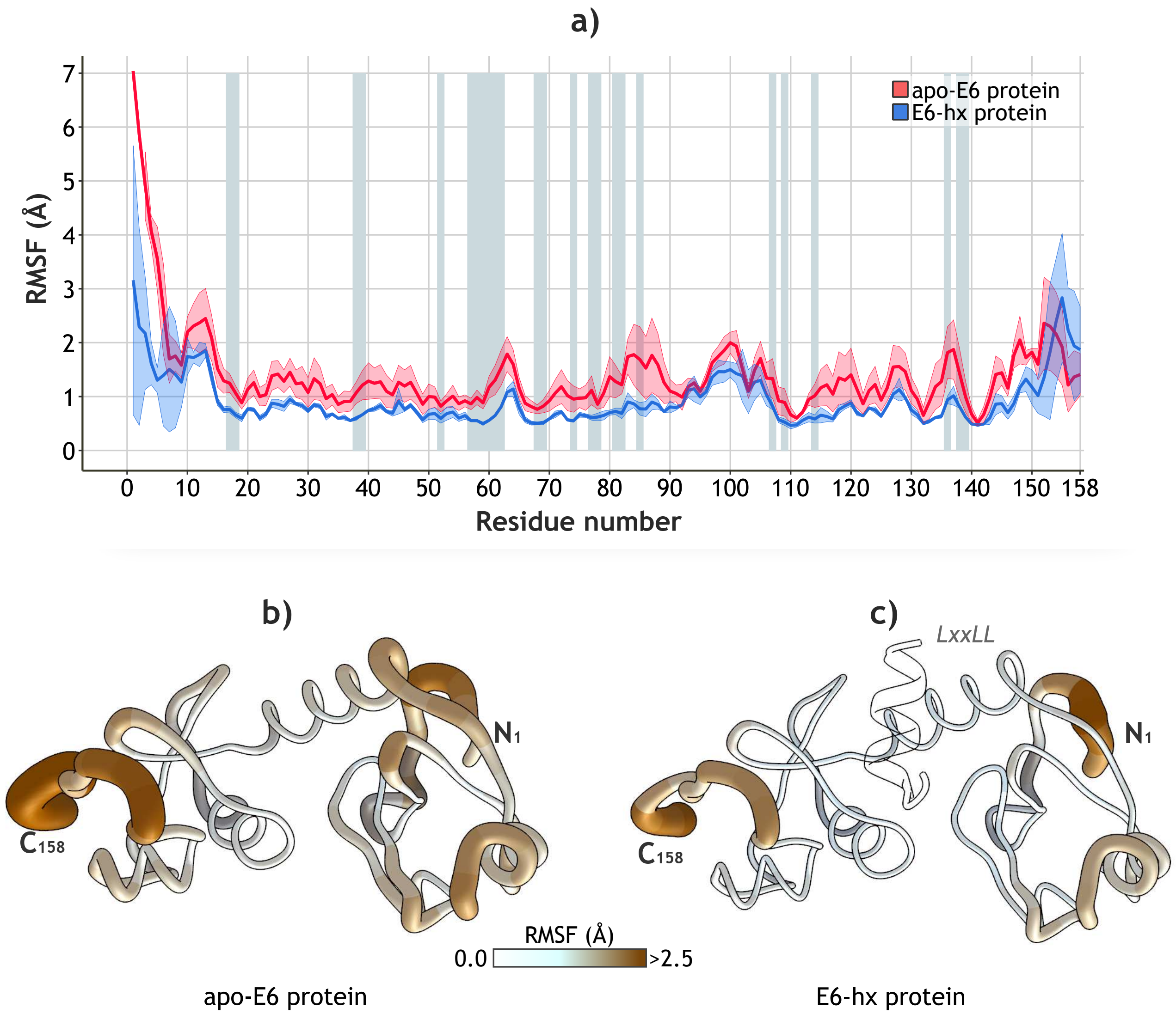

Figure S5: RMSF values of the E6 protein in the apo-E6 and E6-hx systems. a) RMSF values of each residue of the E6 protein (apo-E6 = red, E6-hx = blue). Bold lines correspond to the average RMSF values of the three assays of each system, and semitransparent ribbons display the standard deviations. The gray columns in the background indicate the position of pocket residues. **b)** and **c)** Structural representation of the E6 backbone showing, in a color range (white to brown), the most flexible regions of the protein according to the RMSF value of each residue in each system: apo-E6 and E6-hx.
