# Supplementary material for: Molecular modeling simulation studies reveal new potential inhibitors against HPV E6 protein
Source: PLoS One. 2019 Mar 15;14(3):e0213028. doi: 10.1371/journal.pone.0213028 (PMC6420176; doi:10.1371/journal.pone.0213028)
Supplement: S6 Fig — (PDF) [file pone.0213028.s006.pdf]

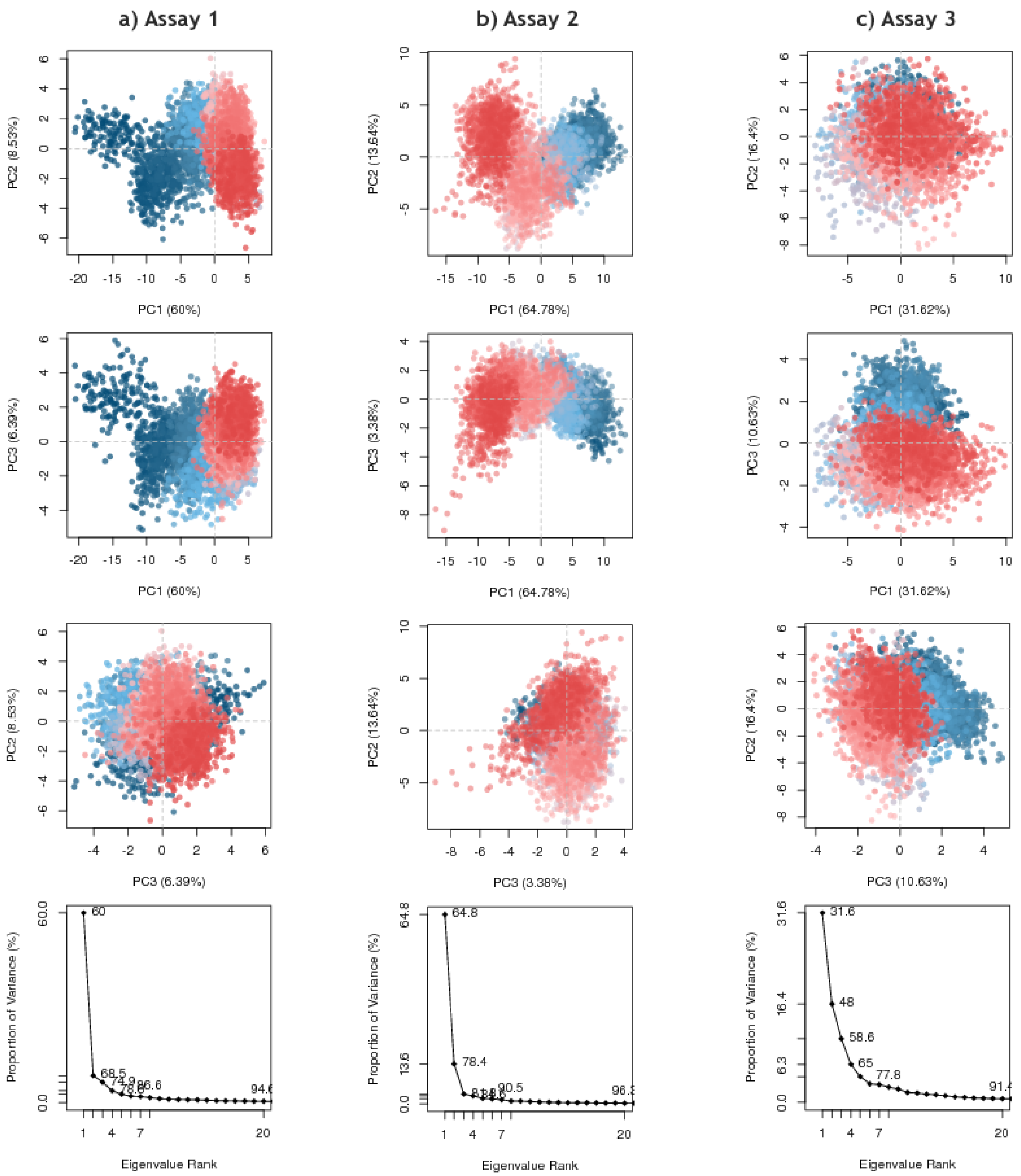

Figure S6: Principal Component Analysis of the three apo-E6 trajectories. Scatter plots and scree plots of the first three PCs (red: 0 ns to blue: 100 ns) from Assay 1 (a), Assay 2 (b) and Assay 3 (c) of apo-E6 system.
