# Supplementary material for: Molecular modeling simulation studies reveal new potential inhibitors against HPV E6 protein
Source: PLoS One. 2019 Mar 15;14(3):e0213028. doi: 10.1371/journal.pone.0213028 (PMC6420176; doi:10.1371/journal.pone.0213028)
Supplement: S7 Fig — (PDF) [file pone.0213028.s007.pdf]

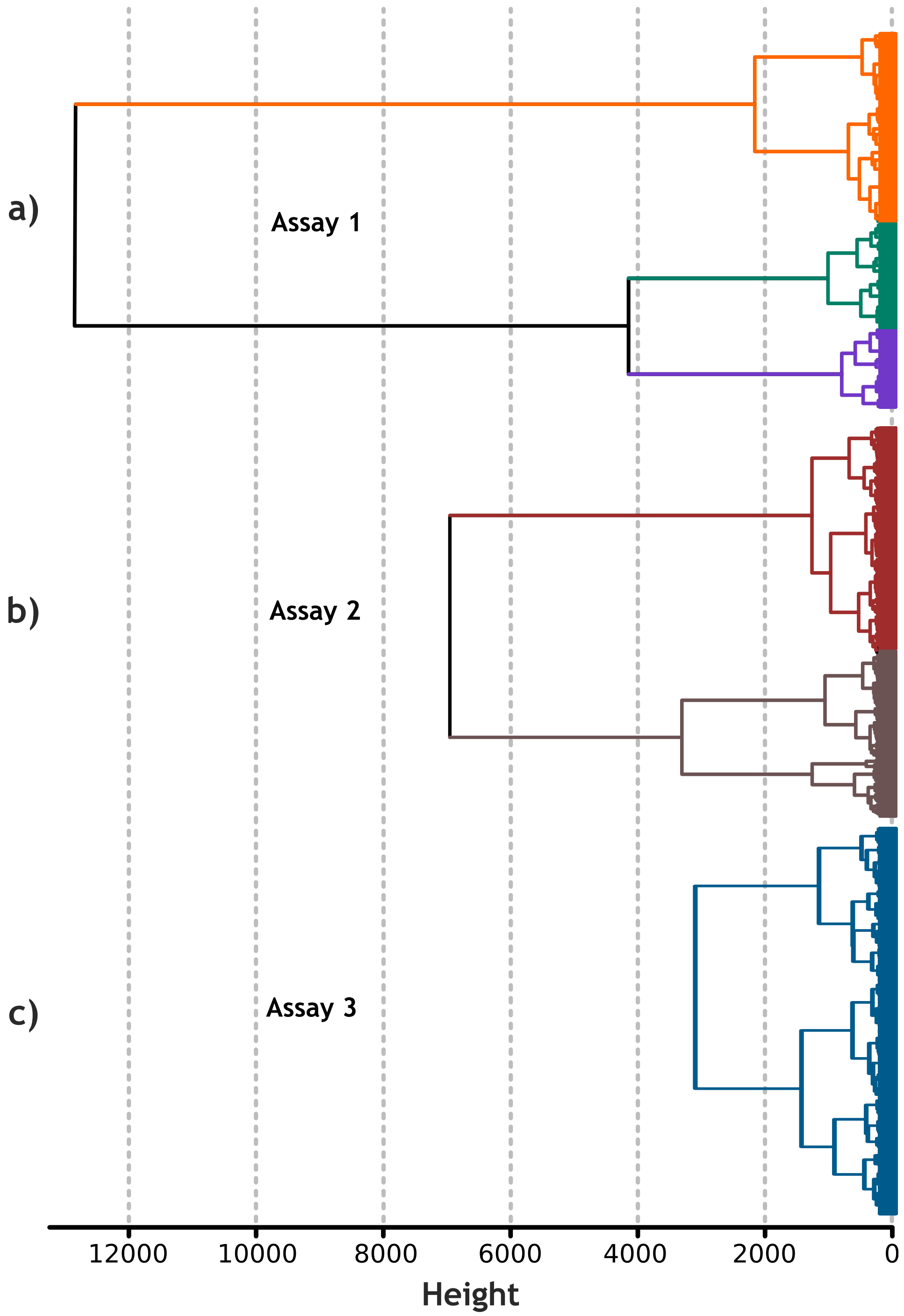

Figure S7: Dendograms resulting from hierarchical clustering analysis of the molecular dynamics trajectories of the apo-E6 system. Each dendogram was generated using Ward's method and euclidean distance metric over the subspace of the three first principal components obtained in each apo-E6 assay. **a)** Assay 1, three groups chosen. **b)** Assay 2, two groups chosen. **c)** Assay 3, one group chosen.
