# Supplementary material for: Molecular modeling simulation studies reveal new potential inhibitors against HPV E6 protein
Source: PLoS One. 2019 Mar 15;14(3):e0213028. doi: 10.1371/journal.pone.0213028 (PMC6420176; doi:10.1371/journal.pone.0213028)
Supplement: S8 Fig — (PDF) [file pone.0213028.s008.pdf]

a)

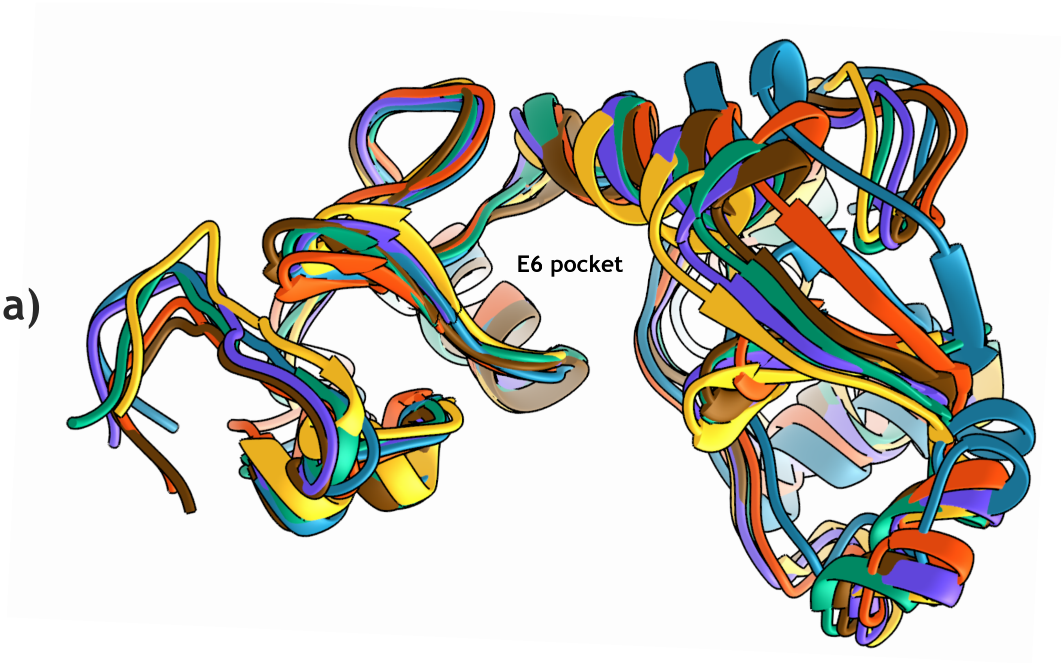

b)

	Homology model P03126	A1:a	A1:b	A1:c	A2:a	A2:b	A3:a	Volumen	Surf. Area
Homology model P03126	0	3.49	3.22	4.21	2.92	1.82	1.35	1,222.6 Å^3	271.3 Å^2
A1:a	3.66	0	0.76	1.08	1.23	2.33	4.03	799.6 Å^3	244.8 Å^2
A1:b	3.65	1.31	0	1.45	0.89	2.08	3.81	862.0 Å^3	277.3 Å^2
A1:c	4.27	1.47	1.49	0	1.92	3.11	4.75	671.0 Å^3	238.7 Å^2
A2:a	3.42	1.2	1.42	2	0	1.79	3.5	904.8 Å^3	297.3 Å^2
A2:b	2.42	2.56	2.61	3.32	2.21	0	2.36	1,073.3 Å^3	308.4 Å^2
A3:a	1.51	4.38	4.46	5.02	4.13	3.05	0	1,204.4 Å^3	305.3 Å^2

RMSD (Å)

Figure S8: Representative conformations of E6 protein. **a)** Superimposed structures of the six conformations selected from apo-E6 trajectories. **b)** RMSD values of each conformer pair, the homology model P03126 is also included. Squares represent the RMSD value of the whole backbone. Circles correspond to the RMSD values of the pocket residues.
