# Supplementary material for: Molecular modeling simulation studies reveal new potential inhibitors against HPV E6 protein
Source: PLoS One. 2019 Mar 15;14(3):e0213028. doi: 10.1371/journal.pone.0213028 (PMC6420176; doi:10.1371/journal.pone.0213028)
Supplement: S9 Fig — (PDF) [file pone.0213028.s009.pdf]

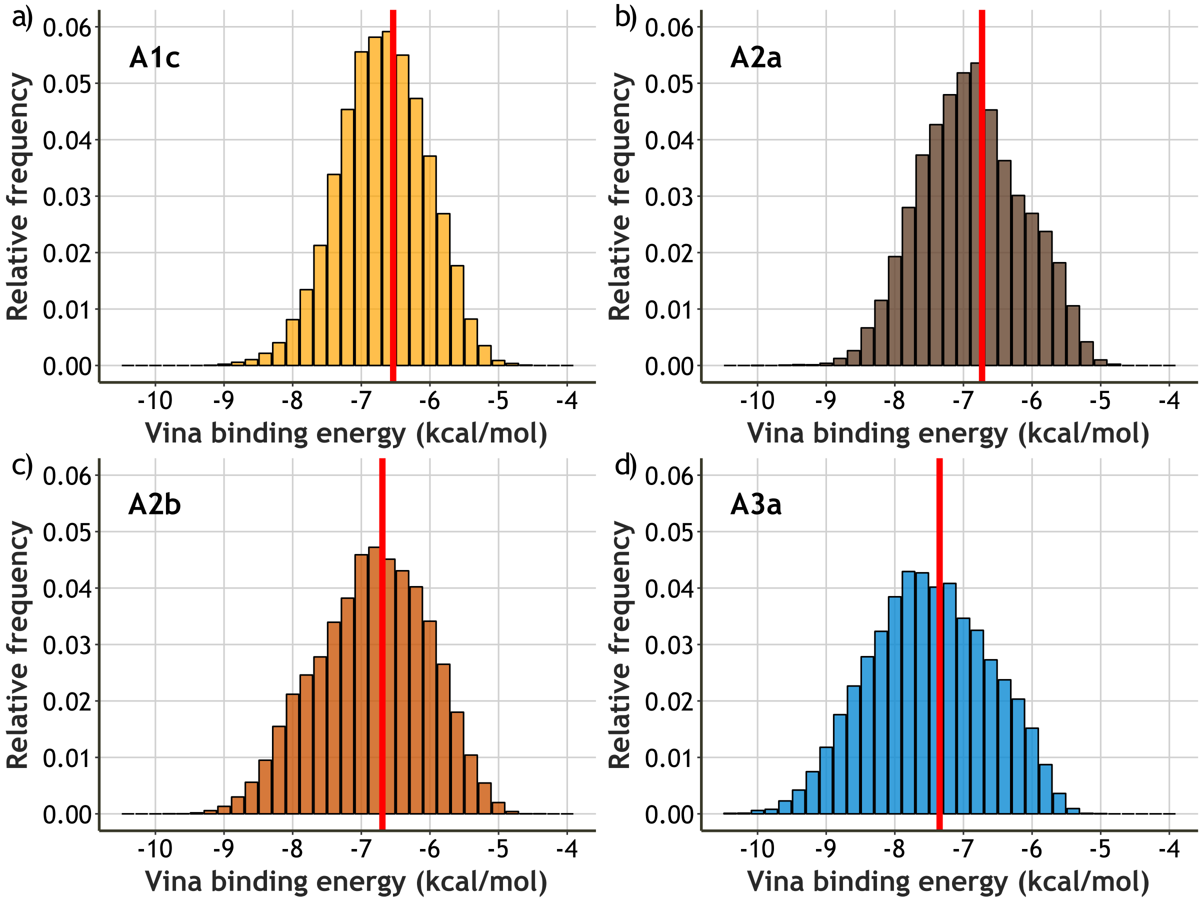

Figure S9: Ensemble-based Docking results performed with Autodock Vina (Vina). Histograms showing the relative frequency of the 19,119 molecules according to the Vina binding energy (kcal/mol) to E6 protein. **a)** Assay 1, conformation c. **b)** Assay 2, conformation a. **c)** Assay 2, conformation b. **d)** Assay 3, conformation a.
