# Supplementary material for: Molecular modeling simulation studies reveal new potential inhibitors against HPV E6 protein
Source: PLoS One. 2019 Mar 15;14(3):e0213028. doi: 10.1371/journal.pone.0213028 (PMC6420176; doi:10.1371/journal.pone.0213028)
Supplement: S10 Fig — (PDF) [file pone.0213028.s010.pdf]

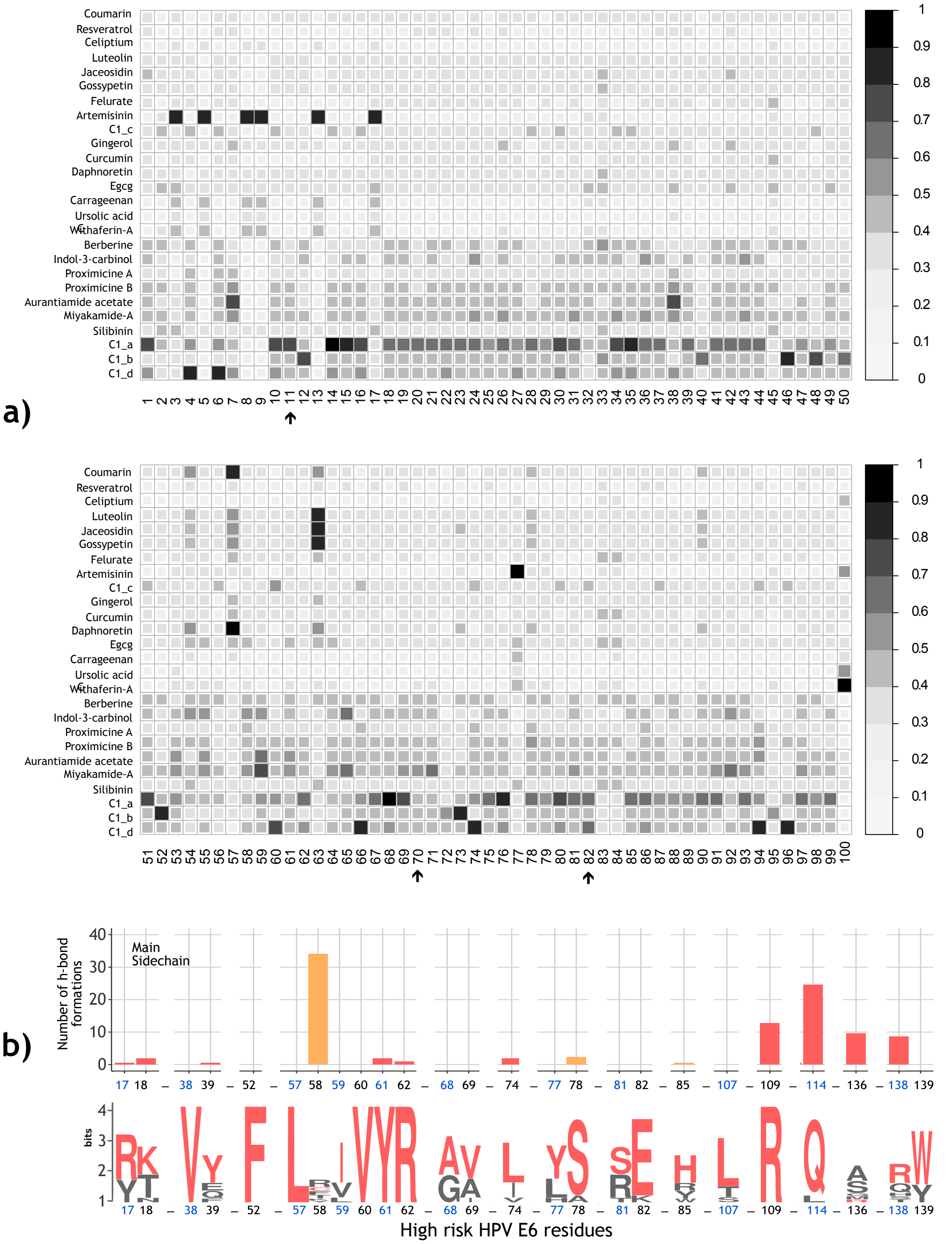

Figure S10: Analysis of the 100 top-ranked ligands according to Autodock 4 score. a) Tanimoto coefficient calculated between the 100 ligands and the 26 reference compounds. The three final candidate compounds chosen for validation by molecular dynamics simulations are indicated by the lower arrows. **b)** Number of hydrogen bonds established between each E6 pocket residue and the the 100 molecules. The sequence logo shows the E6 pocket residue positions of the most prevalent HR HPV types. Red letters indicate the corresponding residue of E6 HPV-16 protein.
