# Supplementary material for: Molecular modeling simulation studies reveal new potential inhibitors against HPV E6 protein
Source: PLoS One. 2019 Mar 15;14(3):e0213028. doi: 10.1371/journal.pone.0213028 (PMC6420176; doi:10.1371/journal.pone.0213028)
Supplement: S11 Fig — (PDF) [file pone.0213028.s011.pdf]

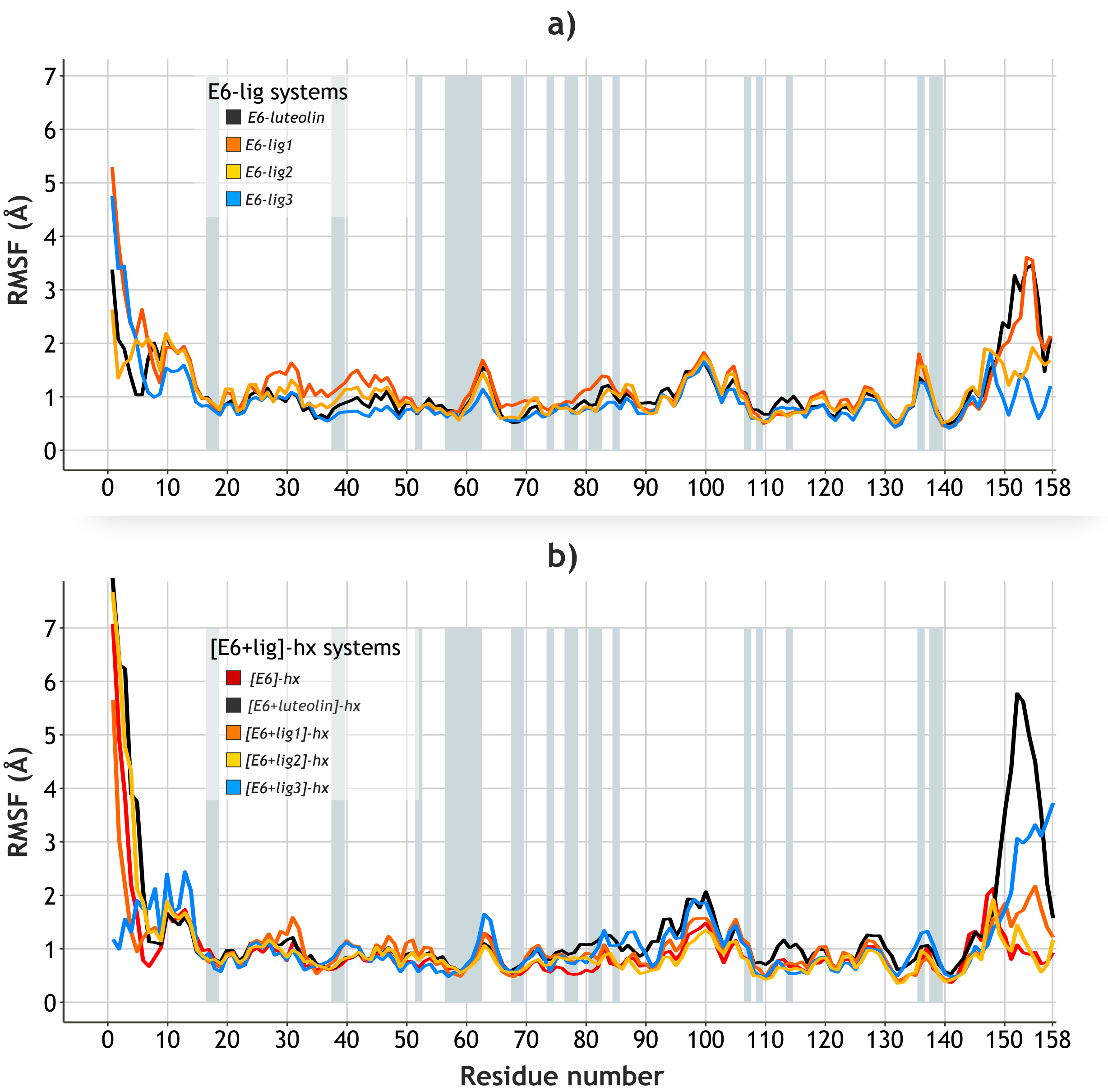

Figure S11: RMSF values of the E6 protein in the E6-lig and [E6+lig]-hx systems. **a)** E6-lig systems, **b)** [E6+lig]-hx systems. The gray columns in the background indicate the position of pocket residues.
