# Supplementary material for: Molecular modeling simulation studies reveal new potential inhibitors against HPV E6 protein
Source: PLoS One. 2019 Mar 15;14(3):e0213028. doi: 10.1371/journal.pone.0213028 (PMC6420176; doi:10.1371/journal.pone.0213028)
Supplement: S13 Fig — (PDF) [file pone.0213028.s013.pdf]

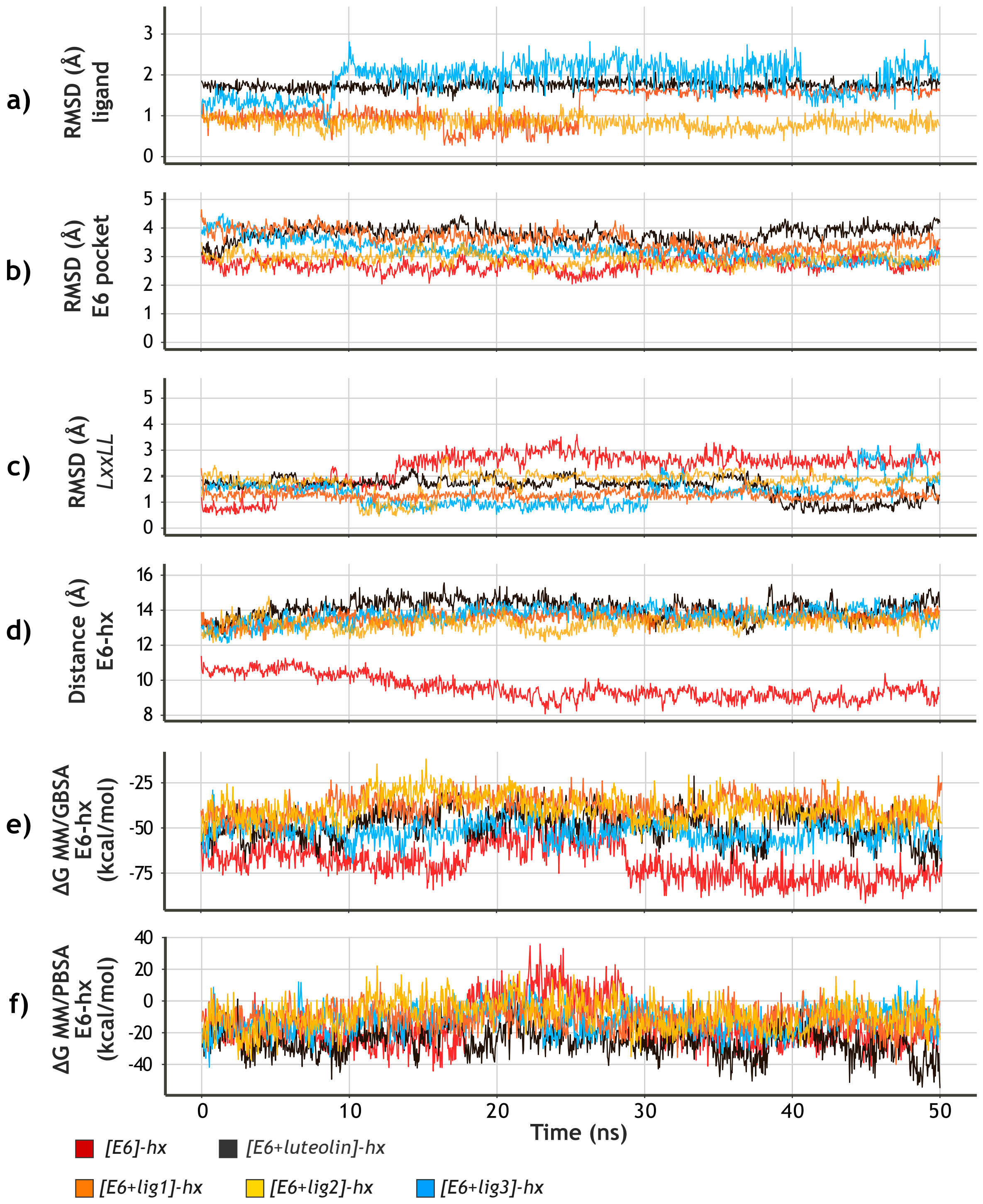

Figure S13: Molecular dynamics of the protein-ligand-*LxxLL* ([E6+lig]-hx) complexes (50ns). **a)** RMSD values of each of the three ligands comparing with its docked pose. **b)** RMSD values of the E6 pocket. **c)** RMSD values of the *LxxLL* motif. **d)** Distance between the center of mass of E6 protein and the center of mass of the *LxxLL* motif. **e)** MM/GBSA ΔG_{bind} values (kcal/mol), and **f)** MM/PBSA ΔG_{bind} values (kcal/mol) between E6-ligand complex and *LxxLL* motif.
