# Supplementary material for: Molecular modeling simulation studies reveal new potential inhibitors against HPV E6 protein
Source: PLoS One. 2019 Mar 15;14(3):e0213028. doi: 10.1371/journal.pone.0213028 (PMC6420176; doi:10.1371/journal.pone.0213028)
Supplement: S15 Fig — (PDF) [file pone.0213028.s015.pdf]

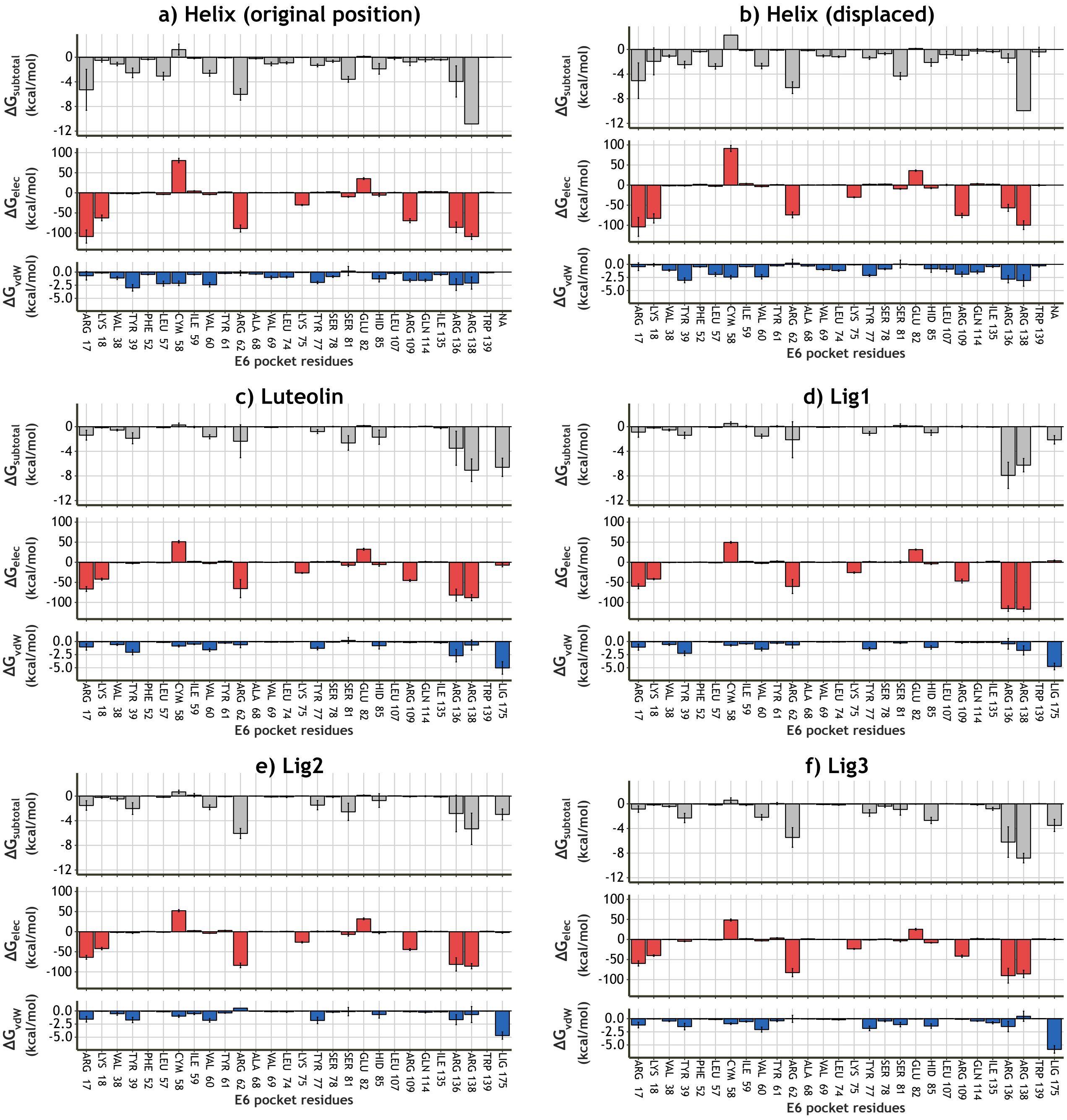

Figure S15: MM/GBSA binding free energy (BFE) decomposition per residue of each of the four [E6+lig]-hx systems, evaluating E6-hx interaction. BFE contribution of each E6 pocket residue ($\Delta G_{subtotal}$) to the E6-*LxxLL* interaction. Electrostatic interaction energies (ΔG_{ele}) and van der Waals interaction energies (ΔG_{vdW}) are also presented. **a)** E6-hx system, **b)** [E6]-hx system, **c)** [E6+luteolin]-hx, **d)** [E6+lig1]-hx, **e)** [E6+lig2]-hx, and **c)** [E6+lig3]-hx.
