# Supplementary material for: Molecular modeling simulation studies reveal new potential inhibitors against HPV E6 protein
Source: PLoS One. 2019 Mar 15;14(3):e0213028. doi: 10.1371/journal.pone.0213028 (PMC6420176; doi:10.1371/journal.pone.0213028)
Supplement: S1 Table — These compounds have shown activity against HPV-positive cells in in vitro assays, and/or against E6 protein in in silico approaches. References corresponding to each molecule are also included. (PDF) [file pone.0213028.s016.pdf]

Table S1: Twenty-six reference compounds identified from the literature. These compounds have shown activity against HPV-positive cells in *in vitro* assays, and/or against E6 protein in *in silico* approaches.

Structure	Name	Structure	Name
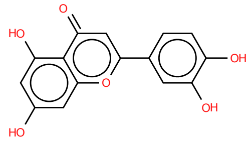	Luteolin<br>ZINC000018185774 [1–4]	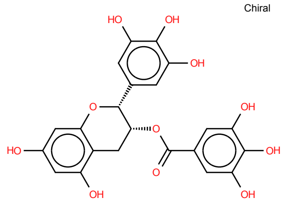	Egcg<br>ZINC000003870412 [5–7]
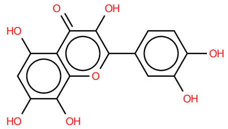	Gossypetin<br>ZINC000006525297 [1]	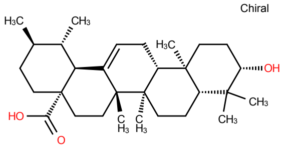	Ursolic-acid<br>ZINC000003978827 [8–10]
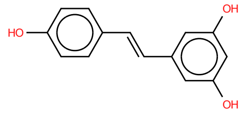	Resveratrol<br>ZINC000000006787 [5,10]	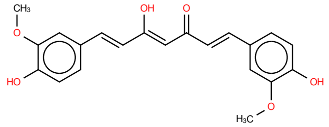	Curcumin<br>ZINC000100067274 [11–15]
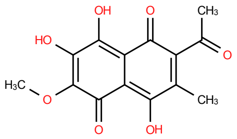	Celiptium<br>ZINC000100089149 [15,16]	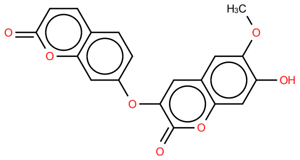	Daphnoretin<br>ZINC000000689683 [15]
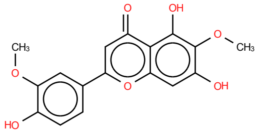	Jaceosidin<br>ZINC000014779854 [4,7,17]	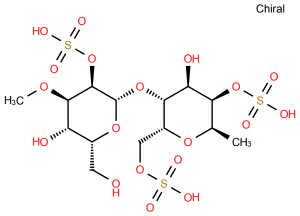	Carrageenan<br>ZINC000064373300 [18]
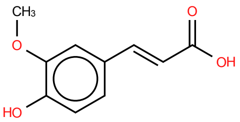	Ferulate<br>ZINC000000058258 [16,19]	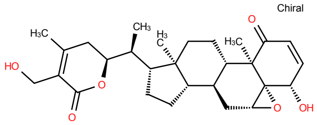	Withaferin-A<br>ZINC000064373300 [10,20,21]
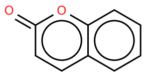	Coumarin<br>ZINC000000074709 [22,23]	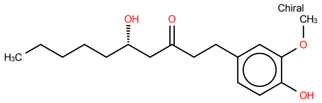	Gingerol<br>ZINC000001531846 [7,24]
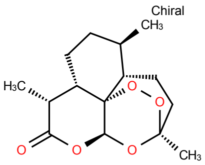	Artemisinin<br>ZINC000008681831 [7,25]	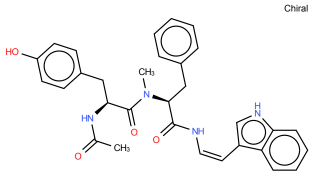	Miyakamide-A<br>ZINC000014594537 [26]

Table S1: Twenty-six reference compounds identified from the literature. These compounds have shown activity against HPV-positive cells in *in vitro* assays, and/or against E6 protein in *in silico* approaches.

Structure	Name	Structure	Name
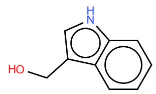	Indol-3-carbinol ZINC000000158743 [27]	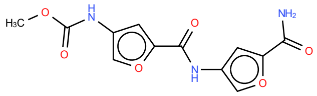	Proximicine-A ZINC000043073751 [26]
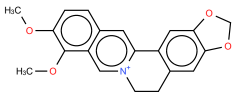	Berberine ZINC000003779067 [28]	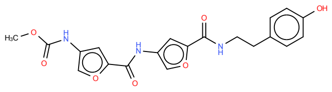	Proximicine-B ZINC000043078506 [26]
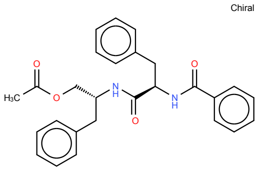	Aurantiamide acetate ZINC000013374322 [26]	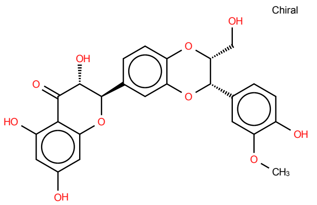	Silibinin ZINC000002033588 [29–31]
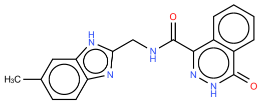	C1-a ZINC000000100126 [10]	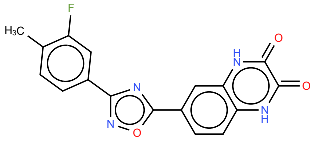	C1-b ZINC000009679272 [10]
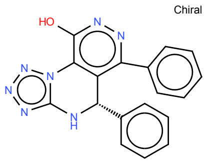	C1-c ZINC000000100126 [10]	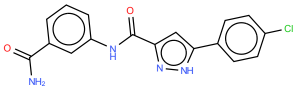	C1-d ZINC000009679272 [10]

References

(1) Malecka, K. A.; Fera, D.; Schultz, D. C.; Hodawadekar, S.; Reichman, M.; Donover, P. S.; Murphy, M. E.; Marmorstein, R. Identification and characterization of small molecule human papillomavirus E6 inhibitors. *ACS Chemical Biology* **2014**, *9*, 1603–1612.

(2) Ham, S.; Kim, K. H.; Kwon, T. H.; Bak, Y.; Lee, D. H.; Song, Y. S.; Park, S. H.; Park, Y. S.; Kim, M. S.; Kang, J. W.; Hong, J. T.; Yoon, D. Y. Luteolin induces intrinsic apoptosis via inhibition of E6/E7 oncogenes and activation of extrinsic and intrinsic signaling pathways in HPV-18-associated cells. *Oncology Reports* **2014**, *31*, 2683–2691.

(3) Shah, M.; Anwar, M. A.; Park, S.; Jafri, S. S.; Choi, S. In silico mechanistic analysis of IRF3 inactivation and high-risk HPV E6 species-dependent drug response. *Scientific reports* **2015**, *5*, 13446.

(4) Cherry, J. J.; Rietz, A.; Malinkevich, A.; Liu, Y.; Xie, M.; Bartolowits, M.; Davisson, V. J.; Baleja, J. D.; Androphy, E. J. Structure based identification and characterization of flavonoids that disrupt human papillomavirus-16 E6 function. *PLoS ONE* **2013**, *8*.

(5) García-Zepeda, S. P.; García-Villa, E.; Díaz-Chávez, J.; Hernández-Pando, R.; Gariglio, P. Resveratrol induces cell death in cervical cancer cells through apoptosis and autophagy. *European journal of cancer prevention : the official journal of the European Cancer Prevention Organisation (ECP)* **2013**, *22*, 577–84.

(6) He, L.; Zhang, E.; Shi, J.; Li, X.; Zhou, K.; Zhang, Q.; Le, A. D.; Tang, X. (-)-Epigallocatechin-3-gallate inhibits human papillomavirus (HPV)-16 oncoprotein-induced angiogenesis in non-small cell lung cancer cells by targeting HIF-1alpha. *Cancer Chemother Pharmacol* **2013**, *71*, 713–725.

(7) Kumar, S.; Jena, L.; Sahoo, M.; Nayak, T.; Mohod, K.; Daf, S.; Varma, A. K. The in Silico Approach to Identifying a Unique Plant-Derived Inhibitor Against E6 and E7 Oncogenic Proteins of High-Risk Human Papillomavirus 16 and 18. *Avicenna Journal of Medical Biochemistry* **2016**, *In Press*.

(8) Kassi, E.; Papoutsi, Z.; Pratsinis, H.; Aligiannis, N.; Manoussakis, M.; Moutsatsou, P. Ursolic acid, a naturally occurring triterpenoid, demonstrates anticancer activity on human prostate cancer cells. *Journal of Cancer Research and Clinical Oncology* **2007**, *133*, 493–500.

(9) Yim, E. K.; Lee, M. J.; Lee, K. H.; Um, S. J.; Park, J. S. Antiproliferative and antiviral mechanisms of ursolic acid and dexamethasone in cervical carcinoma cell lines. *International Journal of Gynecological Cancer* **2006**, *16*, 2023–2031.

(10) Kumar, S.; Jena, L.; Mohod, K.; Daf, S.; Varma, A. K. Virtual Screening for Potential Inhibitors of High-Risk Human Papillomavirus 16 E6 Protein. *Interdisciplinary Sciences: Computational Life Sciences* **2015**, *7*, 136–142.

(11) Divya, C. S.; Pillai, M. R. Antitumor action of curcumin in human papillomavirus associated cells involves downregulation of viral oncogenes, prevention of NFkB and AP-1 translocation, and modulation of apoptosis. *Molecular Carcinogenesis* **2006**, *45*, 320–332.

(12) Maher, D. M.; Bell, M. C.; O'Donnell, E. A.; Gupta, B. K.; Jaggi, M.; Chauhan, S. C. Curcumin suppresses human papillomavirus oncoproteins, restores p53, rb, and ptpn13 proteins and inhibits benzo[a]pyrene-induced upregulation of HPV E7. *Molecular Carcinogenesis* **2011**, *50*, 47–57.

(13) Basu, P.; et al, Clearance of Cervical Human Papillomavirus Infection by Topical Application of Curcumin and Curcumin. *Asian Pacific Journal of Cancer Prevention* **2013**, *14*, 5753–5759.

(14) Singh, A. K.; Misra, K. Human papilloma virus 16 e6 protein as a target for curcuminoids, curcumin conjugates and congeners for chemoprevention of oral and cervical cancers. *Interdisciplinary Sciences: Computational Life Sciences* **2013**, *5*, 112–118.

(15) Mamgain, S.; Sharma, P.; Pathak, R. K.; Baunthiyal, M. Computer aided screening of natural compounds targeting the E6 protein of HPV using molecular docking. *Bioinformation* **2015**, *11*, 236–242.

(16) Kumar, S.; Jena, L.; Sahoo, M.; Kakde, M.; Daf, S.; Varma, A. K. In Silico Docking to Explicate Interface between Plant-Originated Inhibitors and E6 Oncogenic Protein of Highly Threatening Human Papillomavirus 18. *Genomics & Informatics* **2015**, *13*, 60.

(17) Lee, H. G.; Yu, K. A.; Oh, W. K.; Baeg, T. W.; Oh, H. C.; Ahn, J. S.; Jang, W. C.; Kim, J. W.; Lim, J. S.; Choe, Y. K.; Yoon, D. Y. Inhibitory effect of jaceosidin isolated from Artemisia argyi on the function of E6 and E7 oncoproteins of HPV 16. *Journal of Ethnopharmacology* **2005**, *98*, 339–343.

(18) Buck, C. B.; Thompson, C. D.; Roberts, J. N.; Müller, M.; Lowy, D. R.; Schiller, J. T. Carrageenan is a potent inhibitor of papillomavirus infection. *PLoS Pathogens* **2006**, *2*, 0671–0680.

(19) Palasap, A.; Limpaiboon, T.; Boonsiri, P.; Thapphasaraphong, S.; Daduang, S.; Suwannalert, P.; Daduang, J. Cytotoxic effects of phytophenolics from Caesalpinia mimosoides Lamk on cervical carcinoma cell lines through an apoptotic pathway. *Asian Pacific Journal of Cancer Prevention* **2014**, *15*, 449–454.

(20) Park, J. W.; Min, K.-J.; Kim, D. E.; Kwon, T. K. Withaferin A induces apoptosis through the generation of thiol oxidation in human head and neck cancer cells. *International Journal of Molecular Medicine* **2015**, *35*, 247–252.

(21) Munagala, R.; Kausar, H.; Munjal, C.; Gupta, R. C. Withaferin a induces p53-

dependent apoptosis by repression of HPV oncogenes and upregulation of tumor suppressor proteins in human cervical cancer cells. *Carcinogenesis* **2011**, *32*, 1697–1705.

(22) Benci, K.; Mandić, L.; Suhina, T.; Sedić, M.; Klobučar, M.; Pavelić, S. K.; Pavelić, K.; Wittine, K.; Mintas, M. Novel coumarin derivatives containing 1,2,4-triazole, 4,5-dicyanoimidazole and purine moieties: Synthesis and evaluation of their cytostatic activity. *Molecules* **2012**,

(23) Lacy, A. Studies on Coumarins and Coumarin-Related Compounds to Determine their Therapeutic Role in the Treatment of Cancer. *Current Pharmaceutical Design* **2004**,

(24) Kapoor, V.; Aggarwal, S.; Das, S. N. 6-Gingerol Mediates its Anti Tumor Activities in Human Oral and Cervical Cancer Cell Lines through Apoptosis and Cell Cycle Arrest. *Phytotherapy Research* **2016**, *30*, 588–595.

(25) Jansen, F. H.; Adoubi, I.; J, C. K.; T, D. E. C.; Jansen, N.; Tschulakow, A.; Efferth, T. First study of oral Artenimol-R in advanced cervical cancer: clinical benefit, tolerability and tumor markers. *Anticancer research* **2011**, *31*, 4417–4422.

(26) John, G.; Kotadiya, R. In silico approach to identify putative drugs from natural products for human papillomavirus (HPV) which cause cervical cancer. **2015**,

(27) Bell, M. C.; Crowley-Nowick, P.; Bradlow, H. L.; Sepkovic, D. W.; Schmidt-Grimminger, D.; Howell, P.; Mayeaux, E. J.; Tucker, a.; Turbat-Herrera, E. a.; Mathis, J. M. Placebo-controlled trial of indole-3-carbinol in the treatment of CIN. *Gynecologic oncology* **2000**, *78*, 123–9.

(28) Saha, S. K.; Khuda-Bukhsh, A. R. Berberine alters epigenetic modifications, disrupts microtubule network, and modulates HPV-18 E6-E7 oncoproteins by targeting p53 in cervical cancer cell HeLa: A mechanistic study including molecular docking. *European Journal of Pharmacology* **2015**, *744*, 132–146.

(29) Pirouzpanah, M. B.; Sabzichi, M.; Pirouzpanah, S.; Chavoshi, H.; Samadi, N. Silibilin-induces apoptosis in breast cancer cells by modulating p53, p21, bak and bcl-xl pathways. *Asian Pacific Journal of Cancer Prevention* **2015**, *16*, 2087–2092.

(30) Huang, Q.; Wu, L. J.; Tashiro, S.; Onodera, S.; Li, L. H.; Ikejima, T. Silymarin augments human cervical cancer HeLa cell apoptosis via P38/JNK MAPK pathways in serum-free medium. *J Asian Nat Prod Res* **2005**, *7*, 701–709.

(31) Yu, H. C.; Chen, L. J.; Cheng, K. C.; Li, Y. X.; Yeh, C. H.; Cheng, J. T. Silymarin inhibits cervical cancer cell through an increase of phosphatase and tensin homolog. *Phytotherapy Research* **2012**, *26*, 709–715.
