# Supplementary material for: Molecular modeling simulation studies reveal new potential inhibitors against HPV E6 protein
Source: PLoS One. 2019 Mar 15;14(3):e0213028. doi: 10.1371/journal.pone.0213028 (PMC6420176; doi:10.1371/journal.pone.0213028)
Supplement: S2 Table — (PDF) [file pone.0213028.s017.pdf]

Table S2: Number of compounds filtered out for each *QikProp* property.

	Property/Descriptor	Used criteria	Number of discarded molecules	Reference compounds discarded
1)	**Lipinsky rule of five**	≤ 1 (at most one violation)	from 34 804 to 27 674	Carrageenan and Egcg
2)	**Jorgensen rule of three**	≤ 1 (at most one)	from 27 674 to 24 682	Silibinin
3)	**Human oral absorption**	2 or 3 values	from 24 682 to 20 856	Ursolic Acid and Indol-3-carbinol
4)	**Number of *Qikprop* stars**	≤ 1 (at most one descriptor out of range)	from 20 856 to 20 578	Miyakamide-A
5)	**Prediction of dermal penetration**	Values between -8.0 y 1.0	from 20 578 to 19 119	Aurantiamide acetate
