# Supplementary material for: Molecular modeling simulation studies reveal new potential inhibitors against HPV E6 protein
Source: PLoS One. 2019 Mar 15;14(3):e0213028. doi: 10.1371/journal.pone.0213028 (PMC6420176; doi:10.1371/journal.pone.0213028)
Supplement: S3 Table — (PDF) [file pone.0213028.s018.pdf]

Table S3: Spearman ranking correlation between the Vina ligand rankings for each pair of apo-E6 conformations.

	Conformation	A1c	A2a	A2b	A3a
**1)**	**A1c**	1.00	0.78	0.72	0.76
**2)**	**A2a**	0.78	1.00	0.78	0.82
**3)**	**A2b**	0.72	0.78	1.00	0.83
**4)**	**A3a**	0.76	0.82	0.83	1.00
